# Seasonal extreme temperatures and short-term fine particulate matter increases pediatric respiratory healthcare encounters in a sparsely populated region of the intermountain western United States

**DOI:** 10.1186/s12940-024-01082-2

**Published:** 2024-04-15

**Authors:** Erin L. Landguth, Jonathon Knudson, Jon Graham, Ava Orr, Emily A. Coyle, Paul Smith, Erin O. Semmens, Curtis Noonan

**Affiliations:** 1 Center for Population Health Research, School of Public and Community Health Sciences, University of Montana, 32 Campus Drive, Missoula, MT 59812 USA; 2 Mathematical Sciences, University of Montana, Missoula, USA; 3 Pediatric Pulmonology, Community Medical Center, Missoula, MT USA

**Keywords:** Asthma, Case-crossover design, Distributed lag modeling, Environmental health, Hospital discharge data, Lag effects, Lower respiratory tract infections, Montana, PM_2.5_, Rural, Upper respiratory tract infections, Wildfire smoke, Woodfire smoke, Environmental public health, Fine particulate matter air pollution, Respiratory infections, Environmental public health, Fine particulate matter air pollution, Respiratory infections

## Abstract

**Background:**

Western Montana, USA, experiences complex air pollution patterns with predominant exposure sources from summer wildfire smoke and winter wood smoke. In addition, climate change related temperatures events are becoming more extreme and expected to contribute to increases in hospital admissions for a range of health outcomes. Evaluating while accounting for these exposures (air pollution and temperature) that often occur simultaneously and may act synergistically on health is becoming more important.

**Methods:**

We explored short-term exposure to air pollution on children’s respiratory health outcomes and how extreme temperature or seasonal period modify the risk of air pollution-associated healthcare events. The main outcome measure included individual-based address located respiratory-related healthcare visits for three categories: asthma, lower respiratory tract infections (LRTI), and upper respiratory tract infections (URTI) across western Montana for ages 0–17 from 2017–2020. We used a time-stratified, case-crossover analysis with distributed lag models to identify sensitive exposure windows of fine particulate matter (PM_2.5_) lagged from 0 (same-day) to 14 prior-days modified by temperature or season.

**Results:**

For asthma, increases of 1 µg/m^3^ in PM_2.5_ exposure 7–13 days prior a healthcare visit date was associated with increased odds that were magnified during median to colder temperatures and winter periods. For LRTIs, 1 µg/m^3^ increases during 12 days of cumulative PM_2.5_ with peak exposure periods between 6–12 days before healthcare visit date was associated with elevated LRTI events, also heightened in median to colder temperatures but no seasonal effect was observed. For URTIs, 1 unit increases during 13 days of cumulative PM_2.5_ with peak exposure periods between 4–10 days prior event date was associated with greater risk for URTIs visits that were intensified during median to hotter temperatures and spring to summer periods.

**Conclusions:**

Delayed, short-term exposure increases of PM_2.5_ were associated with elevated odds of all three pediatric respiratory healthcare visit categories in a sparsely population area of the inter-Rocky Mountains, USA. PM_2.5_ in colder temperatures tended to increase instances of asthma and LRTIs, while PM_2.5_ during hotter periods increased URTIs.

**Supplementary Information:**

The online version contains supplementary material available at 10.1186/s12940-024-01082-2.

## Background

Less than 1% of the world experiences daily concentrations of fine particulate matter air pollution (< 2.5 µm in aerodynamic diameter; PM_2.5_) that is less than the recommended daily safe levels of less than a daily concentration average of 15 µg / m^3^ [97]. The daily safe thresholds and related policies have been set based on rigorously designed epidemiological cohort and time series studies (e.g., [69, 70, 88]), confirmed through rigorous re-analysis and subsequent studies over the last several decades [6, 44, 45]. PM_2.5_ affects many health outcomes, but of interest in this study, the role of PM_2.5_ in respiratory health is well known for a range of conditions, including upper respiratory tract infections (URTI; e.g., croup; [21], laryngitis; [14], influenza; [62], COVID-19; [41], lower respiratory tract infections (LRTI; e.g., bronchitis; [46], bronchiolitis; [40], pneumonia; [63]), and chronic disorders (e.g., chronic obstructive pulmonary disease; [78], asthma,[32], lung cancer,[23]. Associative impact studies overwhelmingly corroborate a correlative link between respiratory health outcomes and exposure to air pollutants (e.g., [42, 54, 84]), as well as delayed exposures through both short-term (i.e., same day to 1 month,e.g., [28, 93, 96]) or long-term timeframes (greater than 1 month,e.g., [49, 67]). Inhaling PM_2.5_ can produce inflammation and oxidation stress, triggering cellular damage and increasing the risk of respiratory disease [10].

Ambient PM_2.5_ air pollution, particularly in urban and higher-income country settings, has been significantly reduced over the last 40 years [26, 61]. However, in many areas of the world, and specifically for our rural and intermountain study setting of Montana, USA, exposure to PM_2.5_ continues to increase due to residential wood combustion for heat in the winter season and wildfire smoke events during the summer (or wildfire) season. In the 2022 State of the Air report [5], Montana received failing grades for eight counties based on the number of unhealthy and hazardous air-quality days due to severe wildfires and use of residential wood stoves. Regarding wood stoves, Montana ranks second in the USA in the proportion of households that heat with wood fuel (7.4% compared to 1.7% in the USA; [85]. Chemical Mass Balance source apportionment studies have shown that residential wood stoves are the largest source of ambient PM_2.5_ during the winter months (55.5–82%; [86]). Studies evaluating the health impacts associated with residential sources of PM_2.5_ are limited and often suffer from challenges related to sparse populations and uncertain generalizability [64, 77].

The second air quality threat in the mountain west region is smoke from nearby and distant wildfires. Wildfire-specific PM_2.5_ sources are projected to worsen with climate change [26, 65] with no discrimination for jurisdictions and threatening to reverse decades of policy for clean air standards. A growing body of literature is focused on the health effects of PM_2.5_ specifically derived from wildfire smoke. Health impacts from wildfire smoke exposures range from irritation of the eyes and respiratory tract to respiratory morbidity, with growing evidence supporting an association with all-cause mortality [75]. In particular, hospitalizations and emergency department visits related to respiratory infections and preexisting conditions, such as asthma and COPD, are consistently elevated during and shortly following wildfire events [12, 75]. Several factors complicate the evaluation of wildfire exposures and healthcare usage on health outcomes. These include uncertainty in lag effects and potential non-linear response curves that may indicate lower healthcare utilization during extremely high wildfire smoke events, perhaps mediated through behavior changes that are not at play in urban settings where the moderately elevated PM_2.5_ exposures are less recognizable or notable by community members [31].

In parallel, global exposure to extreme temperatures has grown and is expected to worsen with climate change. Extreme temperature events, both cold and hot, are known to be associated with excess mortality and increased hospital admissions for a range of health outcomes [17, 30]. Hotter days in the summer will cause increased levels of illness and death by compromising the body’s ability to regulate its temperature, or by exacerbating health problems. Cold temperatures in the winter can cause blood vessels to constrict, heightening cardiovascular issues, and irritating the airways triggering respiratory problems and lowering immunity. Specifically focusing on the respiratory-related health categories in this study, literature for temperature-associated respiratory health effects are mixed with respect to hot versus cold temperature extremes and depending on the respiratory categories studied. For example, it is well known that cold (and dry) conditions can increase the survival rate of influenza viruses and enhance viral spread (e.g., [55]). A review concluded that both extreme heat and cold could significantly increase the risk of asthma [34], and higher temperatures have been observed to worsen dyspnea, while colder temperature may trigger cough and phlegm symptoms among COPD patients [76].

Multiple rigorous studies observed impacts on health of PM_2.5_ and temperature, but have considered increases in these exposures separately; however, these exposures often occur simultaneously and may act synergistically on health. Differential PM_2.5_ sources across the seasons (i.e., wood stove and industrial emissions in the winter versus wildfire smoke in the summer) compounded with extreme temperature exposures could differentially affect health outcomes. The potential for interactive effects based on these two climate-relevant factors is important as current population risk estimates and corresponding policy recommendations are based largely on epidemiological studies quantifying the effects of PM_2.5_ and temperature considered in isolation. A systematic review of several studies, almost entirely in urban populations, indicate sufficient findings of moderate quality to support synergistic effects for temperature and air pollution [4], although such evidence for pediatric respiratory outcomes is extremely limited [92]. Assessment of these questions in rural communities also is limited, but a recent case crossover study in California for all age cardiorespiratory hospitalization showed strong evidence for a synergistic effect between wildfire specific PM_2.5_ and extreme heat [13].

For the study presented here, we evaluated associations between short-term or delayed fine particulate matter (PM_2.5_) on three children’s respiratory health outcomes assessed at the individual level. We additionally assessed modification of these associations by temperature and season. We focused on a rural and sparsely populated service area in western Montana, USA, from 2017–2020. This area of the inter-Rocky Mountains is experiencing more frequent exceedance of daily air quality standards in the summer due to increases in wildfire smoke events with the largest source of ambient PM_2.5_ in the winter due to residential wood stoves.

## Methods

All analyses were performed with R software (version 4.2; R Development Core Team) including the ‘lme4’ [7], ‘Tidyverse’ [90], and ‘biostat3’ [82] packages.

### Study area, population, and respiratory health outcomes

The study protocol was approved by the Institutional Review Board (IRB) at the University of Montana. Initial study approval was obtained by the University of Montana-Missoula Institutional Review Board on 6 July 2021 (#97–21). Health data were previously collected administrative data; thus, informed consent requirements did not apply.

**Fig. 1:**
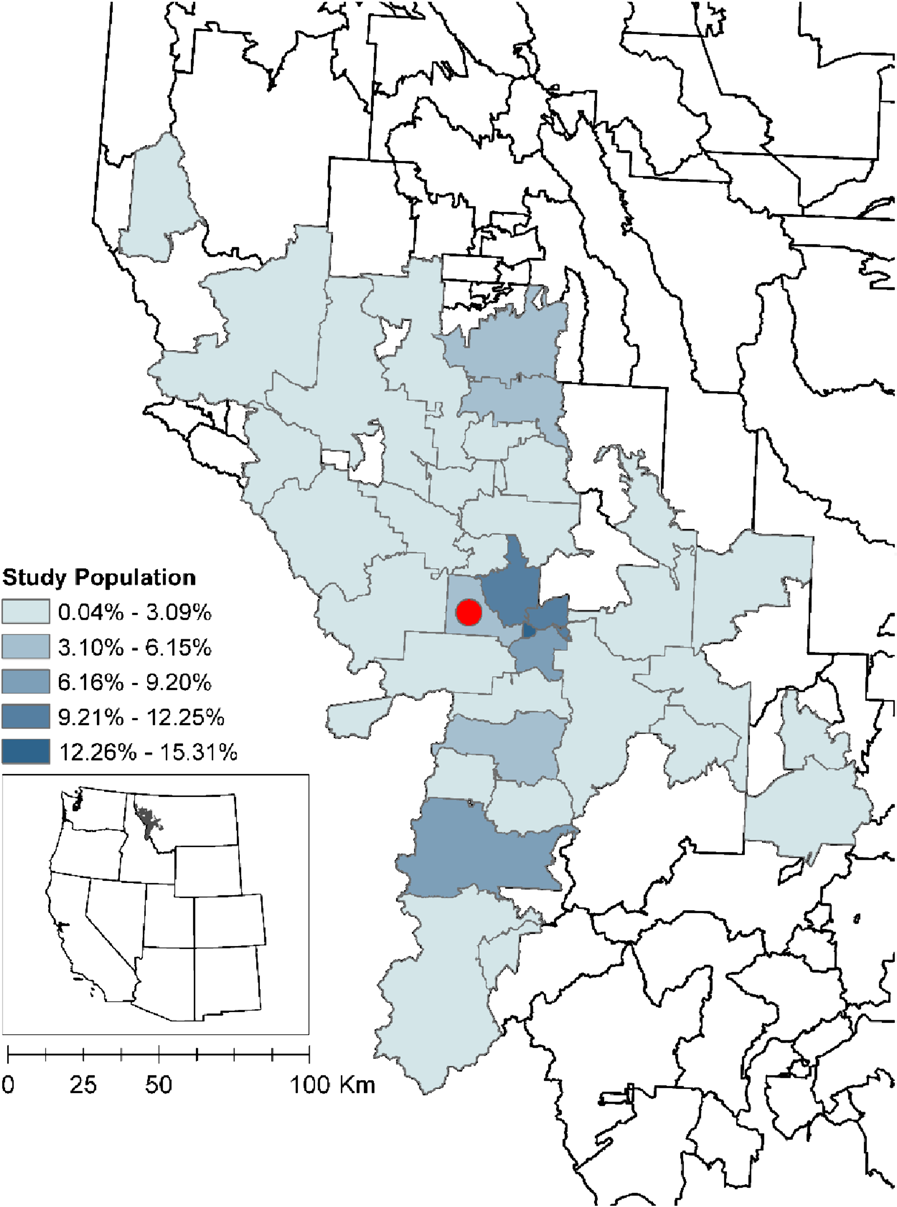
Study area population. 45 ZCTAs in western Montana included in study area symbolized by percent total population. Hospital location (ZCTA = 59804) represented by red circle

*Study area*: For our study, we are focused on western Montana, USA (Fig. 1). The study area covers 45 out of 361 Montana Zip Code Tabulation Areas (ZCTA) across 8 of the 56 counties (Deer Lodge, Granite, Lake, Mineral, Missoula, Powell, Ravalli, and Sanders). The total population within this area was approximately 233,657 in 2020 that includes one small city (Missoula, population total = 73,948) surrounded by sparsely populated areas (US Census Bureau 2020). According to the US Census Bureau’s definition of rurality, this study area is defined as having 72.3% of the population living in rural areas. For context, the US has 19.3% of the population living in rural areas [74]. This region of the inter-Rocky Mountains is experiencing more frequent exceedance of daily air quality standards in the summer months (particularly in July–September [49],) due to increases in wildfire smoke events. The largest source of ambient PM_2.5_ in the winter is due to residential wood stoves [86]. At this northern hemisphere latitude (45–49^o^N), Montana experiences more winter cold months (3.4 on average) than summer warm months (2.8 on average) [27]. Annual average temperatures, including daily minimums, maximums, and averages, have risen across Montana,between 1950 and 2015, with increases ranging from 1.1–1.7 °C [89]. Both wildfire smoke events and extreme temperature conditions are expected to become more common through the twenty-first century.

*Hospital data*: Individual healthcare data at the address-level were collected from 1 December 2017–1 March 2020 for one hospital that predominantly serves the Missoula Valley (Fig. 1) in western Montana, United States, with 10,133 respiratory-related records. These data included nine sources of admissions type: clinic, inpatient, emergency, observation, outreach clinic, preadmission outpatient clinic, professional services, provider clinic, and telemedicine clinic. Data included individuals aged 0–17 with a respiratory-coded infection (see *Case definitions for health outcomes* below and Table 1). A strictly protected health protocol was implemented through data use agreements between the records provider and the University of Montana, where personal identifiers were removed, and residential addresses were geocoded and geomasked. The individual-level data and corresponding spatiotemporal daily PM_2.5_ exposure values were used in case-crossover analyses (see *Case-crossover design and analysis*).

**Table 1:** Case definitions for respiratory infections. ICD-10-CM diagnosis codes for upper respiratory tract infections (URTI), lower respiratory tract infections (LRTI), and asthma

URTI	J00, J01, J01.0, J01.00, J01.01, J01.1, J01.10, J01.11, J01.2, J01.20, J01.21, J01.3, J01.30, J01.31, J01.4, J01.40, J01.41, J01.8, J01.80, J01.81, J01.9, J01.90, J01.91, J02.0, J02.8, J02.9, J03.00, J03.01, J03.80, J03.9, J03.90, J03.91, J04, J04.0, J04.1, J04.10, J04.11, J04.2, J04.3, J04.30, J04.31, J05, J05.0, J05.1, J05.10, J05.11, J06, J06.0, J06.9, J09.X2, J09.X3, J09.X9, J10, J10.0, J10.00, J10.01, J10.08, J10.1, J10.2, J10.8, J10.81, J10.82, J10.83, J10.89, J11, J11.0, J11.00, J11.08, J11.1, J11.2, J11.8, J11.81, J11.82, J11.83, J11.89, J21.0, J21.8, J21.9, H65, H66, H66.9
LRTI	J20, J20.0, J20.1, J20.2, J20.3, J20.4, J20.5, J20.6, J20.7, J20.8, J20.9, J21, J21.0, J21.1, J21.8, J21.9, J09.X1, J09.X2, A37, A37.00, A22.1, A37.01, A37.10, A37.11, A37.80, A37.81, A37.90, A37.91, A48.1, B25.0, J12, J12.0, J12.1, J12.2, J12.3, J12.8, J12.81, J12.82, J12.89, J12.9, J13, J14, J15, J15.0, J15.01, J15.1, J15.2, J15.20, J15.21, J15.211, J15.212, J15.29, J15.3, J15.4, J15.5, J15.6, J15.7, J15.8, J15.9, J16, J16.0, J16.8, J17, J18, J18.0, J18.1, J18.2, J18.8, J18.9, J18, J21, J20.9, R05.1, R05.2
Asthma	J44.0, J44.1, J44.9, J45, J45.2, J45.20, J45.21, J45.22, J45.3, J45.30, J45.31, J45.32, J45.4, J45.40, J45.41, J45.42, J45.5, J45.50, J45.51, J45.52, J45.9, J45.90, J45.901, J45.902, J45.909, J45.99, J45.990, J45.991

**Fig. 2:**
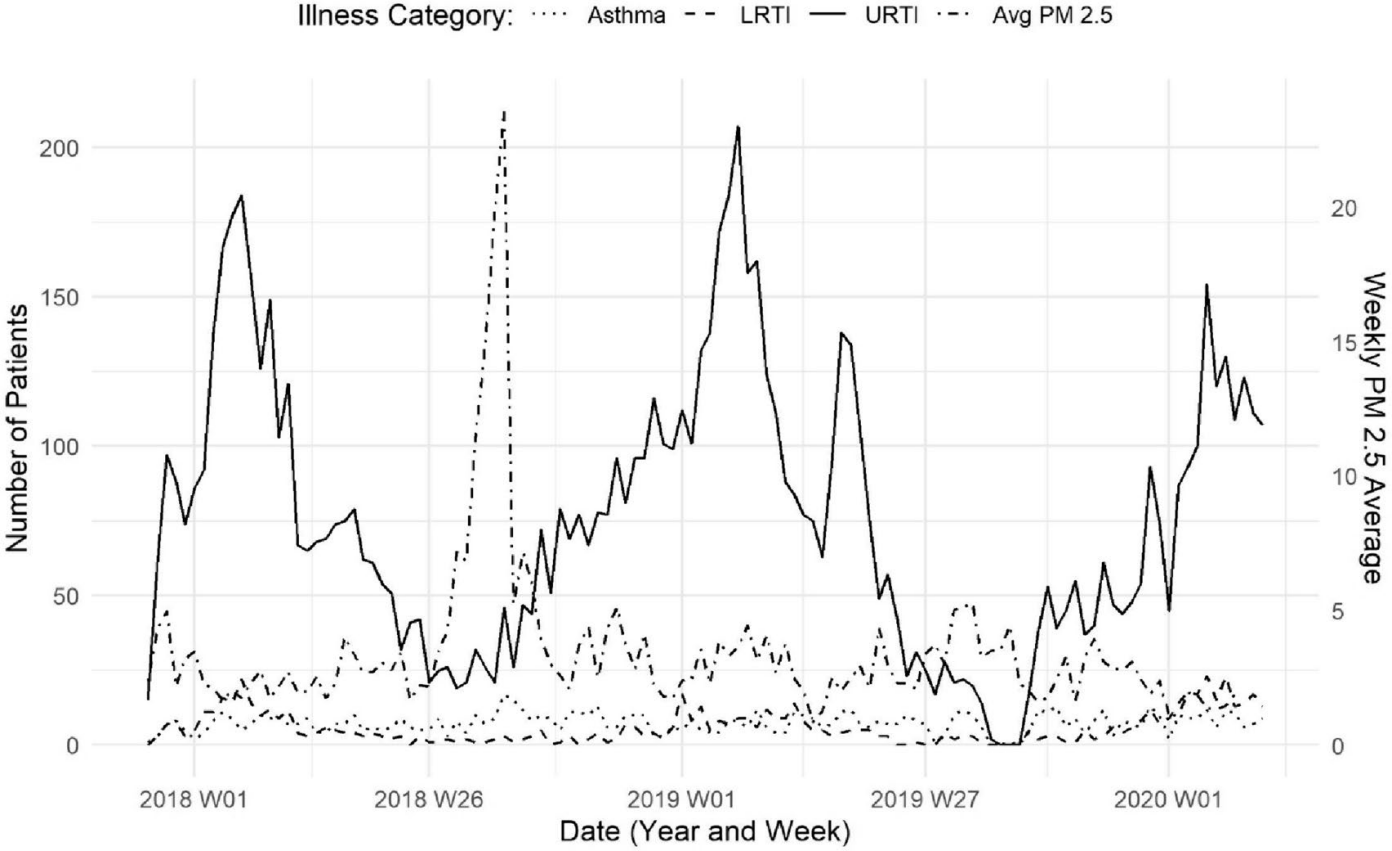
Respiratory healthcare events and PM_2.5_. Related healthcare visits for asthma (dotted line), lower respiratory tract infections (LRTI–dashed line), and upper respiratory tract infections (URTI–solid line), by week, for western Montana residents, aged 0–17. Average PM_2.5_, shown in dot-dashed line for the entire study area, for comparison

*Case definitions for health outcomes*: For this study, cases related to, asthma, lower respiratory tract infections (LRTI), and upper respiratory tract infections (URTI) were first identified using the International Classification of Diseases,10th Revision, Clinical Modification diagnosis codes (ICD-10-CM) and sorted following the case definitions of the Armed Forces Health Surveillance Center (AFHSC 2015) (Table 1). We further identified and split case definitions based on each infection’s upper and lower airway occurrences. Records were classified by condition when a related diagnosis code of interest was found in the primary diagnosis field (first-listed) or any secondary diagnosis field (1–8). Records were selected once for each associated category. For example, records with more than one URTI code were only counted once for the URTI category. If a record had codes for URTI, LRTI, and asthma, the record was counted once in each of the three categories. Healthcare data in the study period and area are shown as total counts for each respiratory category in Fig. 2, along with average weekly PM_2.5_.

### Explanatory variables of interest (see Table 2)

**Table 2:** Explanatory Variable Descriptions. A summary of variables used in modeling is provided. Interactions between these variables were also considered

Variable	Measure Description and Usage	Reference
PM_2.5_	Daily 1-km gridded surface PM_2.5_ estimates were the primary exposure of interest. We extracted PM_2.5_ values at each case event address on date of visit, all 14 days prior to the date of case event for investigating short-term exposure effects, and referent days for the case-cross over design (details provided in text)	[79]
Temperature	Daily 4-km gridded gridMET data were used to extract surface maximum temperature at each case event address on date of visit and corresponding referent days	[1]
Season	At each case event day and corresponding referent days, a categorical variable to indicate the season was used for summer (June–Aug), fall (Sep–Nov), winter (Dec–Feb), and spring (Mar–May)	-

#### PM_2.5_ exposure assessment

The daily time-series dataset of PM_2.5_ surface concentrations was previously developed, and details are reported elsewhere [79]. Briefly, these data were produced from air quality station observations, satellite data, and meteorological data to produce daily 1-km resolution surface PM_2.5_ concentration estimates to explore health outcome impacts of PM_2.5_ across spatiotemporal domains specific to the rural and intermountain western USA. We extracted the daily PM_2.5_ measurements for each case event address location on date of healthcare visit for the case-crossover design, along with PM_2.5_ at that same individual address location for the reference days (see *Case-crossover design and analysis*). Delayed PM_2.5_ exposure effects were then considered through a distributed lag model (DLM) described in more detail in the following *Statistical Modeling* section. In addition, we aggregated the 1-km PM_2.5_ values to the Zip Code-level to explore how population-based / Zip Code-level extractions for each case–control pairings compared in modeled results to individual-based / address-level extracted PM_2.5_ values.

#### Temperature

Temperature is a well-established climate variable that can be correlated with the exposure of interest and also possibly certain respiratory health outcomes of interest to this study (e.g., viral infections such as influenza; [55]). We included daily maximum temperature modeled by gridMET [1] extracted to each individual location, date of healthcare visit, and corresponding reference days for the case-crossover model. Temperature was considered a continuous variable in the analysis,however, we used the 15th, 50th, and 85th percentiles of temperature (cutoffs for colder = -0.7 ^0^C, median = 6.2 ^0^C, and hotter = 20.7 ^0^C) to summarize the estimated marginal effects for the interaction of PM_2.5_ and temperature on the three respiratory outcomes. In addition, we examined the delayed effects of a temperature adjustment from lag day 0 to 6 (i.e., day of case to 6 days prior) where each temperature lag was evaluated in a separate model.

#### Season

In Montana, PM_2.5_ levels spike during summer season due to the primary source of wildfire smoke and during the winter season due to the primary source of wood smoke [86]. We therefore included a categorical season predictor that is assumed to be associated with the exposure of interest and potentially also associated with the respiratory health outcomes of interest. We included a northern hemisphere season as a categorical variable that included summer (June–August), fall (September–November), winter (December–February), and spring (March–May). The modeling described next employed a multiplicative interaction between PM_2.5_ and season to estimate the effect of this relationship on the three respiratory outcomes.

### Statistical modeling

#### Case-crossover design and datasets

We evaluated the synergistic effect of season and temperature extremes with PM_2.5_ predictors on the risk of each respiratory infection outcome (asthma, LRTI, or URTI) using a time-stratified case-crossover design widely used in studies of short-term environmental health exposures (e.g., [80, 87]). Introduced in environmental health studies by Maclure [59], case-crossover designs compare an individual’s (case) exposure immediately prior to or during the defining case event with that same individual’s exposure at different reference (or control) times. This method is attractive because it compares individuals with themselves and controls for time-invariant confounders (age, sex, race, socioeconomic status, and other short timeframe changing health behaviors) and secular trends (long term time trends in exposure or response) by design [57]. Since the seminal Maclure [59] study, several variations on choosing control days to minimize biases have emerged, and convergence to a time-stratified case-crossover design has evolved as the recommended approach for minimizing sources of bias [95]. Thus, we created case-crossover datasets for the 3 respiratory outcomes with paired case events and either 3 or 4 controls. The case event day was defined as the date of healthcare encounter. We then identified matched control days as the same weekdays from other weeks of the same month and year in the same geocoded location of residence (i.e., of the same person). We selected control days both before and after the case day to minimize bias from long-term time trends in temperature and PM_2.5_ [11, 52].

#### Distributed lag modeling

Next, we used distributed lag models (DLM) on the case-crossover datasets. DLMs are a class of models that are used to simultaneously test for lagged measures of exposure (here, PM_2.5_) on an outcome (e.g., [29]. In a DLM, an outcome is regressed on repeated measures of exposures over a proceeding time period. Thus, DLMs were used to determine the sensitive windows of PM_2.5_ exposure on risk of respiratory healthcare encounter. These models used a 14-day lagged length for PM_2.5_ concentration as the exposure variable and main effect variable. This main effect time-lagged variable was then interacted with temperature and season.

To identify sensitive windows within the 14-days of PM_2.5_ exposure and test the effects of short-term PM_2.5_ effects on respiratory health, we investigated how a change of 1 µg / m^3^ in PM_2.5_ for all *k* days from *k* = 0 to 14 days prior to the case (and control) event day. We included 3 variations of the PM_2.5_ lag distribution within the DLM model: (i) Single day – A 1 µg / m^3^ in PM_2.5_ temporary change in a single lagged day. The single day PM_2.5_ lags included the value on the *k*^*th*^ day previous to the case event (e.g., a single day lag 3 corresponds to the PM_2.5_ value that occurred 3 days before the case/control day). (ii) Cumulative days – Cumulative days of PM_2.5_ included the accumulation of PM_2.5_ for all *k* days prior to the case event day (e.g., a cumulative lag 0–14 would include the sum of all 14 days prior to the case/control day), and (iii) Weekly average – Weekly average PM_2.5_ lags included rolling averages of PM_2.5_ over 1-week periods prior to the case event, including the average PM_2.5_ value for 0–6, 1–7, …, 8–14 days prior.

For each respiratory binary response (asthma, LRTI, or URTI) and corresponding case-crossover dataset, we applied a conditional logistic model to estimate the odds ratios for the cumulative effect or the expected difference in the respiratory outcome that is association with a simultaneous 1 µg/m^3^ unit increase in PM_2.5_ at each time point. Given that our modeling also included interactions, the marginal representation of the cumulative effect requires that we fix the values of the other interacting variables. For temperature, we summarized across the 15th, 50th, and 85th percentiles of temperature (colder, median, and hotter, respectively). For season, we summarized across fall, winter, spring, and summer. Modification of the effects of PM_2.5_ on each respiratory health outcome by temperature or season was assessed by including multiplicative interaction terms for PM_2.5_-temperature or PM_2.5_-season. The three-way interaction terms for PM_2.5_-temperature-season were not included due to model instability (low sample sizes). To account for the effects of temperature or season, appropriate linear combinations of coefficients were utilized using the ‘biostat3’ R package [82].

## Results

In summary, we analyzed respiratory healthcare visit data for a sparsely populated region in western Montana, USA. During the study period (1 December 2017 – 1 March 2020), we observed 10,133 respiratory visits among 8,128 unique patients, including 794 asthma, 638 LRTI, and 8,392 URTI. Figure 2 illustrates the weekly case counts and seasonal patterns across the time period studied. Modeled daily PM_2.5_ concentrations within our study area and period ranged from 0.45 to 40.40 µg / m^3^. Summary statistical values for the year 2018 and 2019, respectively, were mean = 4.52 and 3.15, SD = 5.506 and 2.443, median = 2.87 and 2.72, and interquartile range (IQR) = 3.12 and 2.22 µg / m^3^. Approximately 209 of 821 days exceeded the daily average standard of 15 µg / m^3^ (the current WHO 24-h average standard; [88]) in this study area with 26 days in summer, 58 days in fall, 103 days in winter, and 22 days in spring. Notably, 8 of the highest days that exceeded the United States Environmental Protection Agency 24-h standard of 35 µg / m^3^ occurred during August 2018 when a prolonged air pollution event was experienced in the area due to smoke transport from extensive wildfire activity in the western US and Canada.

In what follows, we report the odds ratios for modeled results of each respiratory outcome (asthma, LRTI, URTI). We note upfront that our exploratory model runs that aggregated the 1-km PM_2.5_ values to the Zip Code-level produced significantly smaller effect sizes than what is presented here in the results for the individual-level extracted PM_2.5_ values (Supplementary Fig. 1). In addition, model comparisons between delayed effects of only temperature did not vary across temperature’s time-lagged days 0 to 6 (Supplementary Fig. 2), and the lag day 0 for temperature are present in the results as follows. Finally, PM_2.5_ single day time lagged models were not as consistent as cumulative days and average weekly days, and to ease the viewing of all model combinations, the single day PM_2.5_ time-lagged models are presented in the Additional File 1.

### Asthma

**Fig. 3:**
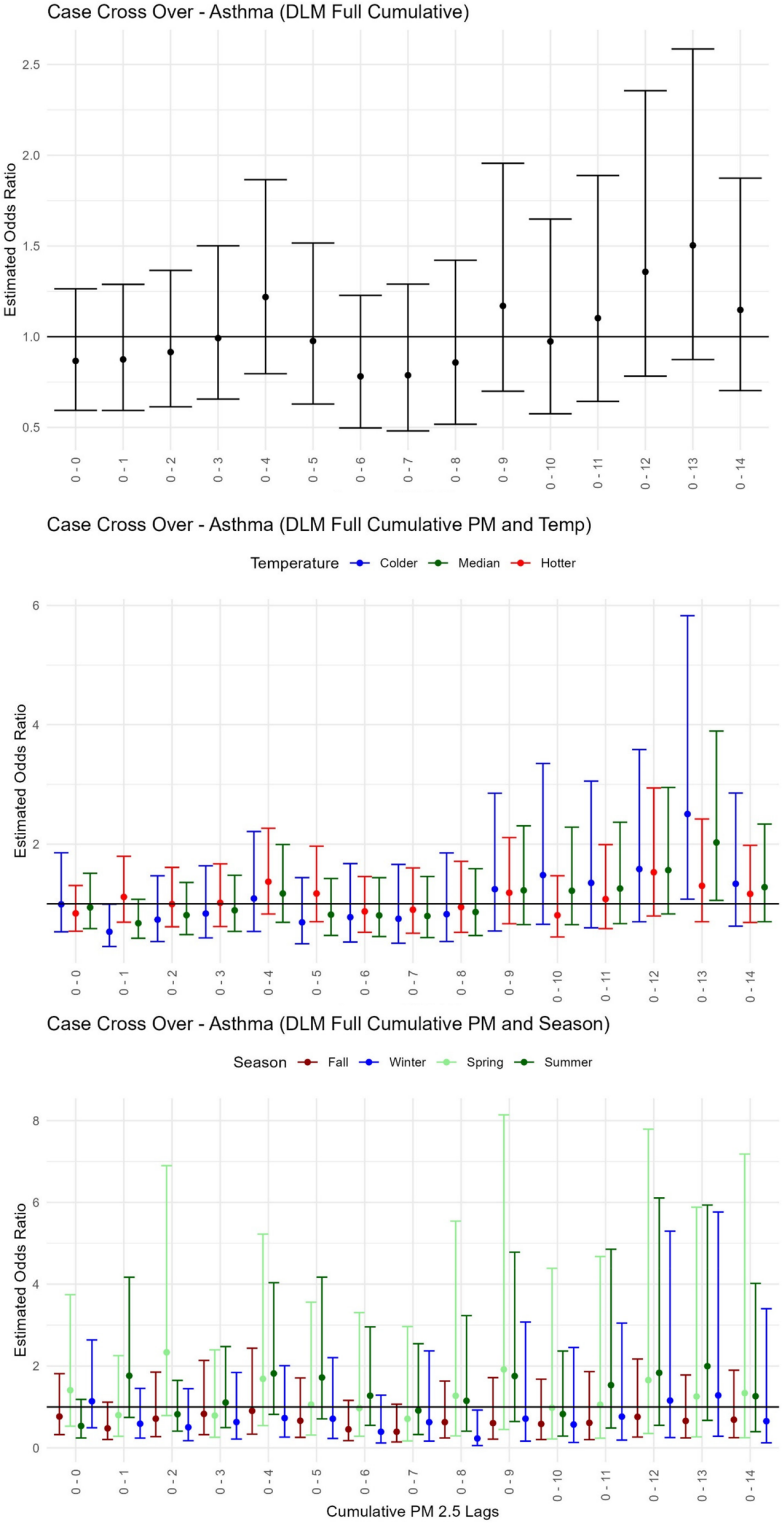
Pediatric asthma health event risk with short-term exposure to PM_2.5_modified by temperature or season. Estimated odds ratios with confidence intervals from the case cross-over distributed lag models using delays for cumulative days of PM_2.5_ for (**A**) the Main PM_2.5_ model–only PM_2.5_, (**B**) the Temperature model–PM_2.5_-Temp summarized across 3 groups for hotter (red), median (green) and colder (blue) temperatures, and (**C**) the Season model–PM_2.5_-Season displayed for 4 groups of Fall (maroon), Winter (blue), Spring (light green), and Summer (green)

All results for the risk of asthma healthcare visits associated with each 1 µg / m^3^ change in PM_2.5_ modified by temperature or season can be found in Table 3A, Fig. 3, and Supplementary Fig. 3, 4 and 5. For the main effect of PM_2.5_, we observed positive associations with asthma healthcare events at weekly average 7–13 days before date of healthcare visit [OR = 1.92, 95% CI: (1.20–3.06); Supplementary Fig. 3]. These associations were elevated in colder temperatures [OR = 3.23, 95% CI: (1.45–7.18)], followed by median temperatures [OR = 2.52, 95% CI: (1.39–4.55)], but no association was observed with PM_2.5_ modified by hotter temperatures (Supplementary Fig. 4). Accumulated PM_2.5_ (0–13 days) also pointed to increased asthma risk in colder to median temperatures (Table 3A, Fig. 3B). Finally, in the winter season and during the same lag of 7–13 days prior to an event (Supplementary Fig. 5), a 1 µg / m^3^ increase in PM_2.5_ was associated with increased odds of these delayed asthma healthcare events [OR = 3.26, 95% CI: (1.07–9.95)]. Notably, one of the only significant single day time-lagged runs was observed in the winter season during elevated PM_2.5_ levels 9 days prior an asthma event [OR = 3.08, 95% CI: (1.18–8.04); Supplementary Fig. 5]. No significant PM_2.5_ effects on asthma during hotter temperatures or other seasons (spring, summer, or fall) were observed.

**Table 3:** Modification of the effect of PM_2.5_ exposure on respiratory health by temperature or season. The odds ratios and corresponding 95% confidence intervals (CI) with *P*-values to estimate the increase in risk of healthcare encounter for each 1 µg/m^3^ increase in PM_2.5_ at the given lagged cumulative days or weekly average for each temperature/season combination and 3 respiratory outcomes: (A) asthma, (B) lower respiratory tract infections (LRTI), and (C) upper respiratory tract infections (URTI). For each respiratory condition, the 3 exposure models are presented for PM_2.5_, PM_2.5_-Temperature (grouped by Colder, Median, and Hotter for display), and PM_2.5_-Season (grouped by Fall, Winter, Spring, and Summer). A single critical window was chosen to display based on the main PM_2.5_ effect model’s largest odds ratio value. For all lagged windows, see Figs. 3, 4 and 5 and Supplementary Figs. 3, 4, 5, 6, 7, 8, 9, 10 and 11. In addition, bolded values indicate an odds ratio with 95% CI that occurred above the baseline risk of 1.0. n was the sample size for model and group where available, noting that temperature was interacted as a continuous variable and doesn’t define groups

(A) Asthma								
Model	Group	Cumulative Days			Weekly Average			n
		Lag	Odds Ratio (CI)	*P*-val	Lag	Odds Ratio (CI)	*P*-val	
PM_2.5_	–	0–13	1.50 (0.87–2.59)	0.140	**7–13**	**1.92 (1.20–3.06)**	**0.006**	794
PM_2.5_ - Temp	Colder	**0–13**	**2.51 (1.07–5.82)**	**0.038**	**7–13**	**3.23 (1.45–7.18)**	**0.004**	–
	Median	**0–13**	**2.03 (1.05–3.89)**	**0.033**	**7–13**	**2.52 (1.39–4.55)**	**0.002**	–
	Hotter	0–13	1.30 (0.69–2.42)	0.406	7–13	1.49 (0.86–2.58)	0.153	–
PM_2.5_ - Season	Fall	0–13	0.66 (0.24–1.78)	0.412	7–13	1.45 (0.57–3.64)	0.426	191
	Winter	0–13	1.28 (0.29–5.76)	0.744	**7–13**	**3.26 (1.07–9.95)**	**0.038**	247
	Spring	0–13	1.25 (0.26–5.88)	0.772	7–13	1.29 (0.33–4.97)	0.709	190
	Summer	0–13	2.00 (0.67–5.94)	0.214	7–13	1.57 (0.60–4.06)	0.357	166
**(B) LRTI**								
Model	Group	Cumulative Days			Weekly Average			n
		Lag	Odds Ratio (CI)	*P*-val	Lag	Odds Ratio (CI)	*P*-val	
PM_2.5_	**–**	**0–12**	**2.42 (1.13–5.20)**	**0.022**	**6–12**	**2.36 (1.28–4.32)**	**0.006**	638
PM_2.5_ - Temp	Colder	0–12	2.09 (0.81–5.32)	0.123	**6–12**	**2.82 (1.23–6.48)**	**0.014**	–
	Median	**0–12**	**2.33 (1.06–5.12)**	**0.034**	**6–12**	**2.55 (1.35–4.81)**	**0.004**	–
	Hotter	0–12	2.94 (0.91–9.50)	0.070	6–12	2.06 (0.72–5.82)	0.175	–
PM_2.5_ - Season	Fall	0–12	1.46 (0.18–11.4)	0.716	6–12	3.02 (0.52–17.4)	0.218	61*
	Winter	0–12	1.36 (0.36–5.09)	0.646	6–12	1.49 (0.60–3.68)	0.389	374
	Spring	0–12	1.65 (0.27–9.81)	0.579	6–12	3.74 (0.87–16.1)	0.076	169
	Summer	0–12	1.89 (0.06–63.6)	0.723	6–12	0.05 (0.00–3.56)	0.172	34*
**(C) URTI**								
Model	Group	Cumulative Days			Weekly Average			n
		Lag	Odds Ratio (CI)	*P*-val	Lag	Odds Ratio (CI)	*P*-val	
PM_2.5_	**–**	**0–13**	**1.37 (1.12–1.65)**	**0.001**	**4–10**	**1.32 (1.12–1.55)**	** < 0.001**	8,392
PM_2.5_ - Temp	Colder	0–13	1.10 (0.86–1.40)	0.435	4–10	1.22 (0.98–1.52)	0.075	–
	Median	**0–13**	**1.30 (1.06–1.59)**	**0.010**	**4–10**	**1.27 (1.07–1.50)**	**0.005**	–
	Hotter	**0–13**	**1.84 (1.41–2.41)**	** < 0.001**	**4–10**	**1.38 (1.08–1.77)**	**0.009**	–
PM_2.5_ - Season	Fall	0–13	0.69 (0.46–1.00)	0.053	4–10	0.85 (0.59–1.19)	0.345	1,266
	Winter	0–13	1.22 (0.86–1.74)	0.259	4–10	1.10 (0.84–1.44)	0.467	4,224
	Spring	**0–13**	**3.06 (1.89–4.95)**	** < 0.001**	**4–10**	**3.28 (2.18–4.91)**	** < 0.001**	2,254
	Summer	**0–13**	**3.35 (1.85–6.04)**	** < 0.001**	4–10	1.38 (0.85–2.22)	0.191	648

* unstable estimates with *n* < 100

### LRTI

**Fig. 4:**
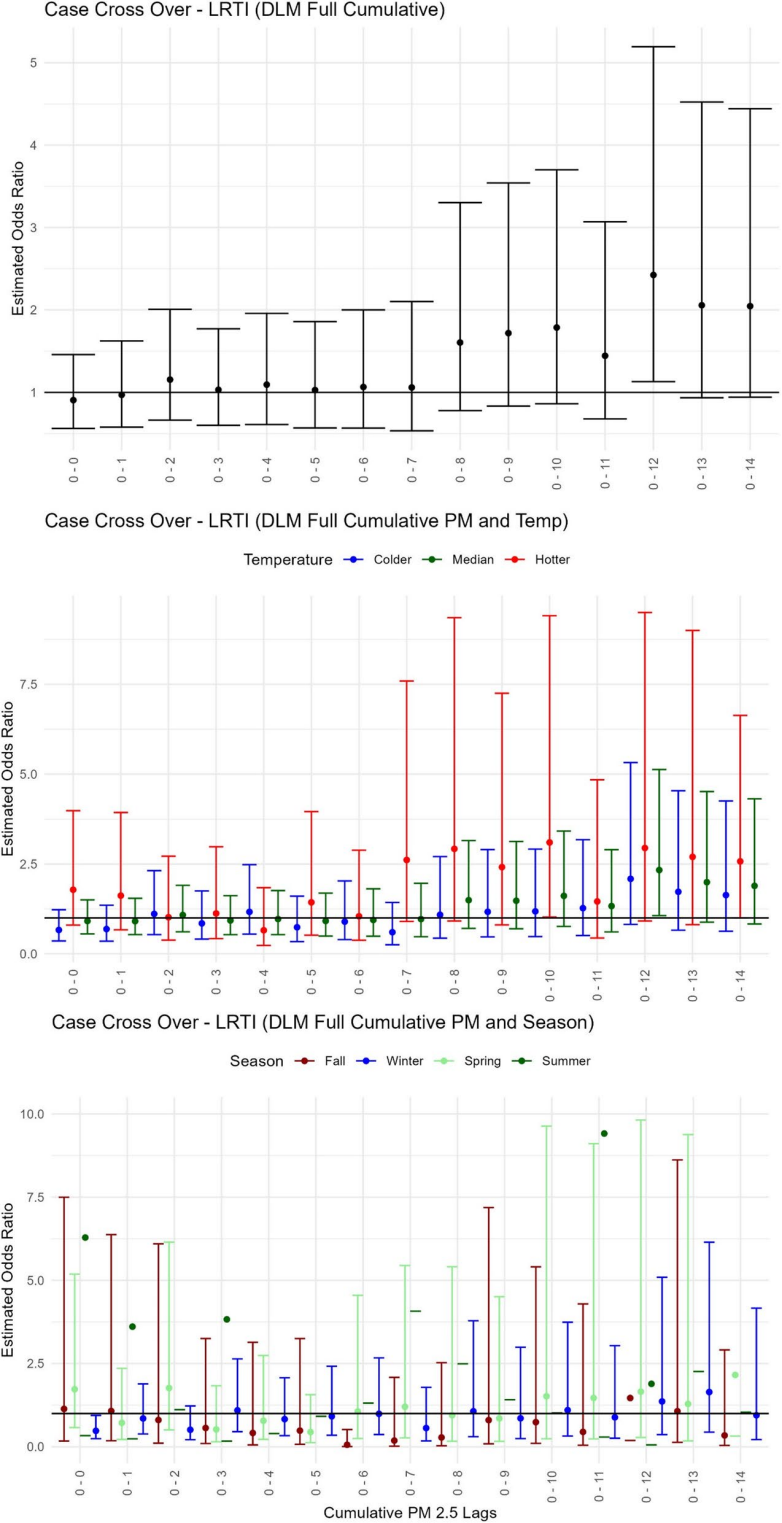
Pediatric lower respiratory tract infection health event risk with short-term exposure to PM_2.5_modified by temperature or season. Estimated odds ratios with confidence intervals from the case cross-over distributed lag models using delays for cumulative days of PM_2.5_ for (**A**) the Main PM_2.5_ model–only PM_2.5_, (**B**) the Temperature model–PM_2.5_-Temp summarized across 3 groups for hotter (red), median (green) and colder (blue) temperatures, and (**C**) the Season model–PM_2.5_-Season displayed for 4 groups of Fall (maroon), Winter (blue), Spring (light green), and Summer (green)

All results for the risk of children’s LRTI healthcare visits associated with each 1 µg / m^3^ change in PM_2.5_ modified by temperature or season can be found in Table 3B, Fig. 4, and Supplementary Fig. 6, 7 and 8. LRTI had the lowest sample size of the respiratory health outcome categories studied here (*n* = 638), resulting in unstable odds estimates for some modeled groups. However, of the LRTI models that had the larger sample sizes (> 100), the PM_2.5_ only model was associated with elevated LRTI healthcare events after an increase of 12 days of accumulating PM_2.5_ [OR = 2.42, 95% CI: (1.13–5.20); Fig. 4A] with a peak exposure period at an average 6–12 days prior to healthcare visit date [OR = 2.36, 95% CI: (1.28–4.32); Table 3B, Supplementary Fig. 6]. In colder and median temperatures during an increase in PM_2.5_ within the same average 6–12 day lag, LRTI healthcare encounters also increased [for colder OR = 2.52, 95% CI: (1.23–6.48), for median OR = 2.55, 95% CI: (1.35–4.81); Supplementary Fig. 7]. Likewise, in median temperatures and after an increase of 12 days of accumulating PM_2.5_, LRTI events also increased [OR = 2.33, 95% CI: (1.05–3.89); Fig. 4B). No significant PM_2.5_ effects on LRTI during hotter temperatures or seasons were observed (Fig. 4C, Supplementary Fig. 8).

### URTI

**Fig. 5:**
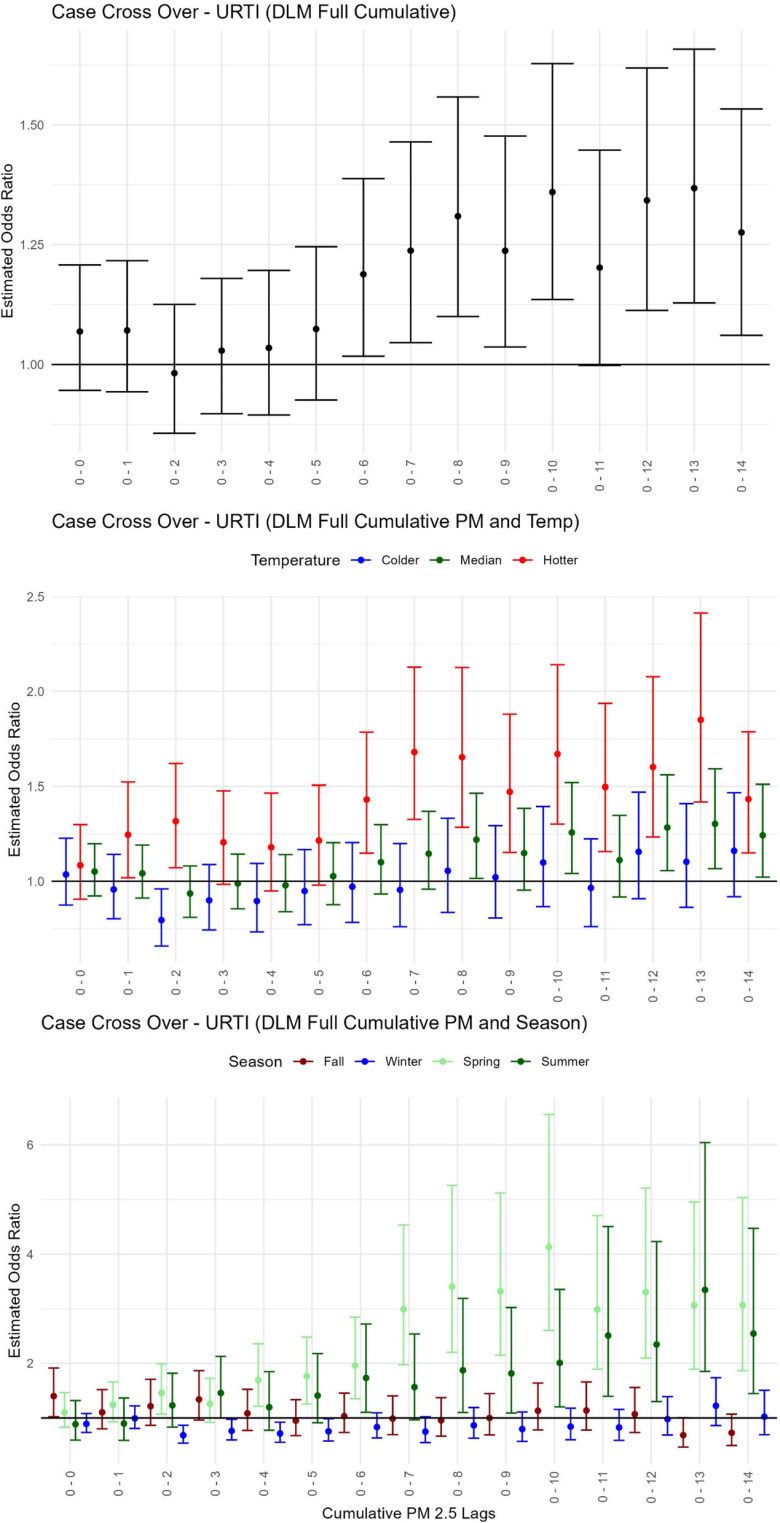
Pediatric upper respiratory tract infection health event risk with short-term exposure to PM_2.5_modified by temperature or season. Estimated odds ratios with confidence intervals from the case cross-over distributed lag models using delays for cumulative days of PM_2.5_ for (**A**) the Main PM_2.5_ model–only PM_2.5_, (**B**) the Temperature model–PM_2.5_-Temp summarized across 3 groups for hotter (red), median (green) and colder (blue) temperatures, and (**C**) the Season model–PM_2.5_-Season displayed for 4 groups of Fall (maroon), Winter (blue), Spring (light green), and Summer (green)

All results for the risk of children’s URTI healthcare events associated with each 1 µg / m^3^ change in PM_2.5_ modified by temperature or season can be found in Table 3C, Fig. 5, and Supplementary Fig. 9, 10 and 11. For the main effect PM_2.5_ only model, increased odds for URTI healthcare events were observed beyond 6 cumulative days of PM_2.5_ with the highest exposure occurring in 13 cumulative days prior visits [OR = 1.37, 95% CI: (1.12–1.65); Fig. 5A] and peak exposure observed during the average window of 4–10 days prior to healthcare visit [OR = 1.32, 95% CI: (1.12–1.55); Supplementary Fig. 9). The higher frequency of URTI outcomes, relative to asthma and LRTI outcomes, allowed for consistent findings of interactive effects by temperature and season and indicated that PM_2.5_ effects were present in hotter rather than colder conditions (Table 3C, Fig. 5B, Supplementary Fig. 10). At cumulative lag (0–13 days), summer and spring periods yielded the highest PM_2.5_ associations with increased odds of URTI healthcare events (for summer OR = 3.35, 95% CI: (1.85–6.04), for spring OR = 3.06, 95% CI: (1.89–4.95); Table 3C, Fig. 5C).

## Discussion

We found that delayed short-term increases in PM_2.5_ air pollution were positively associated with children’s respiratory related healthcare visits and events for a patient population in western Montana, USA. These effects were found for categories of respiratory related visits of asthma (peak effect at lag of 7–13 days), LRTI (peak effect after 12 accumulated days), and URTI (peak effect after 13 accumulated days). These links between increased respiratory risk and increased short-term PM_2.5_ are well established and consistent with past findings. While, in general, consistency of findings implies an association between increased respiratory risk and increased PM_2.5_, some discrepancies between these studies are worth mentioning, including differences in reported observed length of the lag effect and size of effects (discussed more below). Additionally, a newer contribution from our study is that the PM_2.5_ impacts varied by temperature and season, and across respiratory categories all highlighted in the next sections.

### Asthma and PM_2.5_ exposure

Numerous studies link air pollution to asthma. Several reviews have highlighted this connection, specifically for exacerbating existing asthma, but also with an increase of new-onset asthma [32, 83]. A meta-analysis of 84 studies including children, adults, or both found that outdoor air pollutants were associated with an increased risk of asthma exacerbations at lag 0–1 days [36] . The same study also conducted age-based subgroup analyses of children (0–14) and adults (> 14) and found children with asthma were more susceptible to outdoor air pollution [36]. However, various other time-series studies using air pollutants have observed a lag effect with varying results from 0–5 days [33, 39, 51, 58, 68, 72] to 6–7 days [15].  Our study, using the DLM, places the PM_2.5_ associated increased risk in children’s asthma events on the higher delayed end of these studies at a weekly average lag of 7–13 days.

### Asthma and seasonal extreme temperature effects

Our study indicated the highest risk for asthma healthcare visits with increased PM_2.5_ occurred only during colder periods – both cold temperatures and winter periods. Of course, above the 45 ^0^N parallel, these two factors for colder temperatures and winter season are in no doubt, conflated. However, very cold and dry or very hot and humid climate conditions have been shown to exacerbate asthma conditions [16, 25, 48]. An animal model demonstrated that high and low temperatures can aggravate airway inflammation in mice suggesting that asthmatics are more at-risk during exposures to high and low temperature extremes [20]. A recent review found that extreme cold exposures were associated with an increased risk of asthma by 19.77% [34]. Seasonal effects on asthma are inconclusive most likely because a range of temperature conditions have been shown to affect asthma risk. However, increased asthma risk has been observed in only fall and winter seasons [81].

### LRTI and PM_2.5_ exposure

In this study, LRTI encounters for children increased with elevated PM_2.5_ for 12 cumulative days and peaked at a weekly average lag of 6–12 days. Studies on this category of respiratory infections or specific infections within this category (e.g., bronchitis or pneumonia) vary in their findings. To discuss a few, numbers of acute lower respiratory infections for young children in Utah, USA, were found to increase after 1 week of increased PM_2.5_ and peak after 3 weeks of an increased exposure [35], while a similar study and results from Korea found acute lower respiratory infection hospitalizations to be associated with an increase in the 7-day running average of PM_2.5_ [66]. Zhu et al. [99] did not find a significant effect of short-term PM_2.5_ on childhood lower respiratory diseases in China, but did observe the effect with other air pollutants (PM_10_, NO_2_, and SO_2_). In New York, USA, increases in PM_2.5_ from the previous 7 days were found to be associated with hospital visits for culture-negative pneumonia and bacterial pneumonia [18]. To further illustrate variability in results, a meta-analysis review of short-term exposure to PM_2.5_ and pneumonia-related hospitalizations found variable results across study populations, where elderly subgroups showed an increased risk ratio with unclear lag effects and younger patients did not have a significant increase in visits [43].

### LRTI and seasonal extreme temperature effects

Our study showed the highest risk estimates for LRTI as a function of PM_2.5_ during colder temperatures but insufficient sample size to assess any seasonal PM_2.5_ mediated effects. These results are in line with past studies showing LRTI to be a significant cause of hospitalizations, morbidity, and mortality worldwide with seasonal climate factors being associated with a higher probability of infection [24]. For example, Alvaro-Meca et al. [3] found that LRTI hospital visits were more frequent during lower temperatures. And Mäkinen et al. [60] demonstrated that cold temperatures were associated with increased occurrences of LRTI and a decrease in temperature preceded the onset of infections.

### URTI and PM_2.5_ exposure

URTIs have also been extensively studied and linked to air pollutants. Here, we found a positive association between PM_2.5_ and children’s URTI healthcare events with the highest response at 13 days of accumulated PM_2.5_ with a peak at an average of 4–10 days prior to an event. As with asthma and LRTI, past research has shown that study population, study region, methodology, and type of upper respiratory tract infection can produce variations in the length of the delayed short-term effects. For example, in Beijing, China, a positive association between PM_2.5_ and increased influenza cases suggested a 1–2 month delayed response [53]. In Hefei, China, increasing concentrates of most all pollutants at lag days 3–6 were associated with increased URTI in children aged 0–14 years [56], while in Suzhou City, China, PM_2.5_ showed a significant association with these infections in children under 3 years old with a lag of 3 weeks [98]. In Kenya, a 2-week delayed response in children’s URTI from PM_2.5_ exposure was observed [50]. In Poland, moderate exposure to air pollution over 12 weeks was associated with an increased risk of URTI in children aged 3–12 years [73].

### URTI and seasonal extreme temperature effects

Our study showed relationships between increased risk of URTI healthcare events after elevated PM_2.5_ during hotter periods. Elevated levels of PM_2.5_ accumulated across 13 days (with peak at an average 4–10 day lag) during hotter temperatures or during the summer/spring season yielded the highest risk of children’s URTI healthcare events. In general, URTI are thought to be more common in colder temperatures because colder exposure impairs nasal antiviral immunity [22, 37]. Viral infectious diseases affecting the upper tract way, such as influenza, have strong seasonal effects in winter temperate regions and are associated with colder (and dryer) conditions [71]. However, not all URTI spike in winter months in northern temperate sites, and others, like enterovirus and parainfluenza virus, can occur in summer months, and respiratory syncytial virus can occur earlier than influenza in fall months [47]. Rhinoviruses and adenoviruses can circulate throughout the year with occasional peaks in autumn and winter for rhinoviruses and early spring for adenoviruses [19, 38]. In summary, most respiratory viruses follow a seasonal pattern but not all URTI are viruses, and some factors can increase the incidence of URTI, like mass crowding [2], and, as was observed in this study, air pollution.

### On large effect sizes

We observed effect sizes substantially larger than what is commonly reported in the literature, and this difference warrants further comment. Our analyses found a 1 µg / m^3^ increase in ambient PM_2.5_ was associated with 2- or threefold increases in the rates of healthcare visits for pediatric respiratory conditions, whereas many observational studies find much lower effect estimates (e.g., 1.05-fold increases). The analysis presented here is at the address-level allowing for more precise individual-level PM_2.5_ estimates. These larger effect sizes can occur when we transition from using PM_2.5_ estimates averaged over municipalities or jurisdictional boundaries to more precise PM_2.5_ estimates at a place of residence within those boundaries. Individual exposures within a jurisdictional boundary can vary greatly and result in large differences in individual exposure values, especially for small populated rural and intermountain areas. Evidence of this hypothesis is shown in our post-hoc analysis when exposure is assessed at the Zip Code level and yields effect sizes similar to what is observed in studies that aggregate to large spatial units (see Supplementary Fig. 1). Although we cannot say with certainty that finer scale exposure assessment explains our large effect sizes, these higher resolution exposure estimates may be important, particularly in areas in which PM_2.5_ may vary markedly over space.

### Limitations

Air pollution case-crossover studies for small and rural populations are not without limitations. First, we acknowledge the study’s sample size. These data cover 821 days with an average of 12.8 events per day among all three outcomes (1.06, 0.84, and 10.9 events for asthma, LRTI, and URTI, respectively). It has been suggested in simulation studies of pollution effects that thousands of observation days with an average of tens of events per day are needed [91]. In addition, it is known that small sample sizes result in more random error, possibly biasing results. Second, we acknowledge that unmeasured time-variant factors might have provided additional confounding influence and could possibly impact estimates [8, 9]. To the degree that these factors occur at the individual level, e.g., immunity or vaccination status, the impact is likely to be negligible given the case-crossover design [11]. Third, we acknowledge that error in diagnostic coding is quite possible within these data. Some cases may not be accurately categorized, and it is possible that such coding errors could be differential with respect to season. Fourth, the assessment of exposure could be subject to measurement (and modeling) error, especially in a rural, sparsely populated study area with only a limited number of fixed air quality monitors contributing to the estimates of PM_2.5_ [79]. However, we expect this error would have results in attenuated effect estimates [94]. Finally, it is important to remember that these individual-level healthcare events are a combination of inpatient and outpatient visits. Direct comparison to only one data type might not be applicable.

## Conclusions

Western Montana, USA, is a sparsely populated region of the inter-Rocky Mountains with complex air pollution patterns. This region is experiencing more frequent exceedance of daily air quality standards due to increases in wildfire smoke events during their summer/wildfire season months. However, the region also experiences elevated levels of PM_2.5_ during winter months from wood stove use with complex mountain meteorology and inversion effects. Here, we explored short-term PM_2.5_ effects on three pediatric respiratory health outcomes (asthma, LRTI, and URTI) and how other factors, such as extreme temperature or seasonal period, modify the risk of air pollution-associated hospital visits. We found associations between elevated PM_2.5_ exposures and healthcare visits for all respiratory categories. We found interaction effects with extreme temperatures and during high impacted PM_2.5_ seasons. We found increased risk for asthma and LRTI associated with elevated levels of PM_2.5_ in colder temperatures, while increased risk for URTI associated with elevated levels of PM_2.5_ in hotter periods. Finally, we observed very large effect sizes that we hypothesized are a result of higher resolution estimates of exposure, emphasizing the importance of fine-scale exposure measurement, particularly in areas in which PM_2.5_ may vary markedly over space.

Finally, communities in the western US will experience increases in morbidity and mortality related to higher frequency of extreme temperature and wildfire events [89]. At present, policy and public health messaging related to air pollution and extreme temperatures flow through different agency pathways. For example, extreme cold and heat advisories often occur in advance based on local National Weather Service forecasting, and air quality advisories often occur in real-time according to EPA-based Air Quality Index measures. Communities at risk of wildfire smoke exposures and extreme temperature events need locally-informed guidance, integrated strategies that address these compound risks, and communication approaches that include local knowledge and trusted sources. Local communities will be increasingly burdened with developing and sustaining strategies for adaptation and resilience to climate change, but we lack rigorous and reproducible models for such strategies, particularly as applicable to rural communities in the mountain west that additionally suffer from limited infrastructure that can be leveraged for mitigation.

### Supplementary Information

**Supplementary Material 1.**

## Data Availability

The dataset(s) supporting the conclusions of this study are confidential with the exception of the environmental variables that are publicly available datasets. The code that supported the findings of this study are available from the corresponding author, ELL, upon reasonable request.

## Abbreviations

LRTI: Lower respiratory tract infections
MT: Montana, USA
PM_2.5_: Fine particulate matter air pollution (< 2.5 µm in aerodynamic diameter)
URTI: Upper respiratory tract infections
