# Supplementary material for: Seasonal extreme temperatures and short-term fine particulate matter increases pediatric respiratory healthcare encounters in a sparsely populated region of the intermountain western United States
Source: Environ Health. 2024 Apr 15;23:40. doi: 10.1186/s12940-024-01082-2 (PMC11017546; doi:10.1186/s12940-024-01082-2)

**Supplementary Materials**

Seasonal extreme temperatures and short-term fine particulate matter increases pediatric respiratory healthcare events in a sparsely populated region of the intermountain western United States

Erin L. Landguth, Jonathon Knudson, Jon Graham, Ava Orr, Emily A. Coyle, Paul Smith, Erin O. Semmens, Curtis Noonan

**Contents:**

**Supplemental Figure 1.** Individual-based / address-level data aggregated to the population-based / Zip Code-level.

**Supplemental Figure 2.** Temperature lagged model comparisons.

**Supplemental Figure 3.** Asthma and PM_2.5_.

**Supplemental Figure 4.** Asthma and PM_2.5_ modified by temperature.

**Supplemental Figure 5.** Asthma and PM_2.5_ modified by season.

**Supplemental Figure 6.** LRTI and PM_2.5_.

**Supplemental Figure 7.** LRTI and PM_2.5_ modified by temperature.

**Supplemental Figure 8.** LRTI and PM_2.5_ modified by season.

**Supplemental Figure 9.** URTI and PM_2.5_.

**Supplemental Figure 10.** URTI and PM_2.5_ modified by temperature.

**Supplemental Figure 11.** URTI and PM_2.5_ modified by season.

**Supplemental Figure 1 - Individual-based / address-level data aggregated to the population-based / Zip Code-level.** Here, models used the same case-crossover datasets, but with PM_2.5_ values extracted at the Zip Code polygons in the study area – that is, if 2 individuals lived in the same Zip Code, they most likely received the same PM_2.5_ values for case and control pairings. A conditional logistic regression model was used to estimate odds ratios for each single day lag of PM_2.5_ (non-DLM model) on (A) asthma, (B) LRTI, and (C) URTI. Note that estimate odds ratios are smaller in these population-based PM_2.5_ results (maximum OR = 1.05) than for modeled runs that used the individual-based PM_2.5_ data (comparable but DLM runs can be observed in Supplemental Figure 3, 6, and 9: maximum OR = 1.67).

**(A)
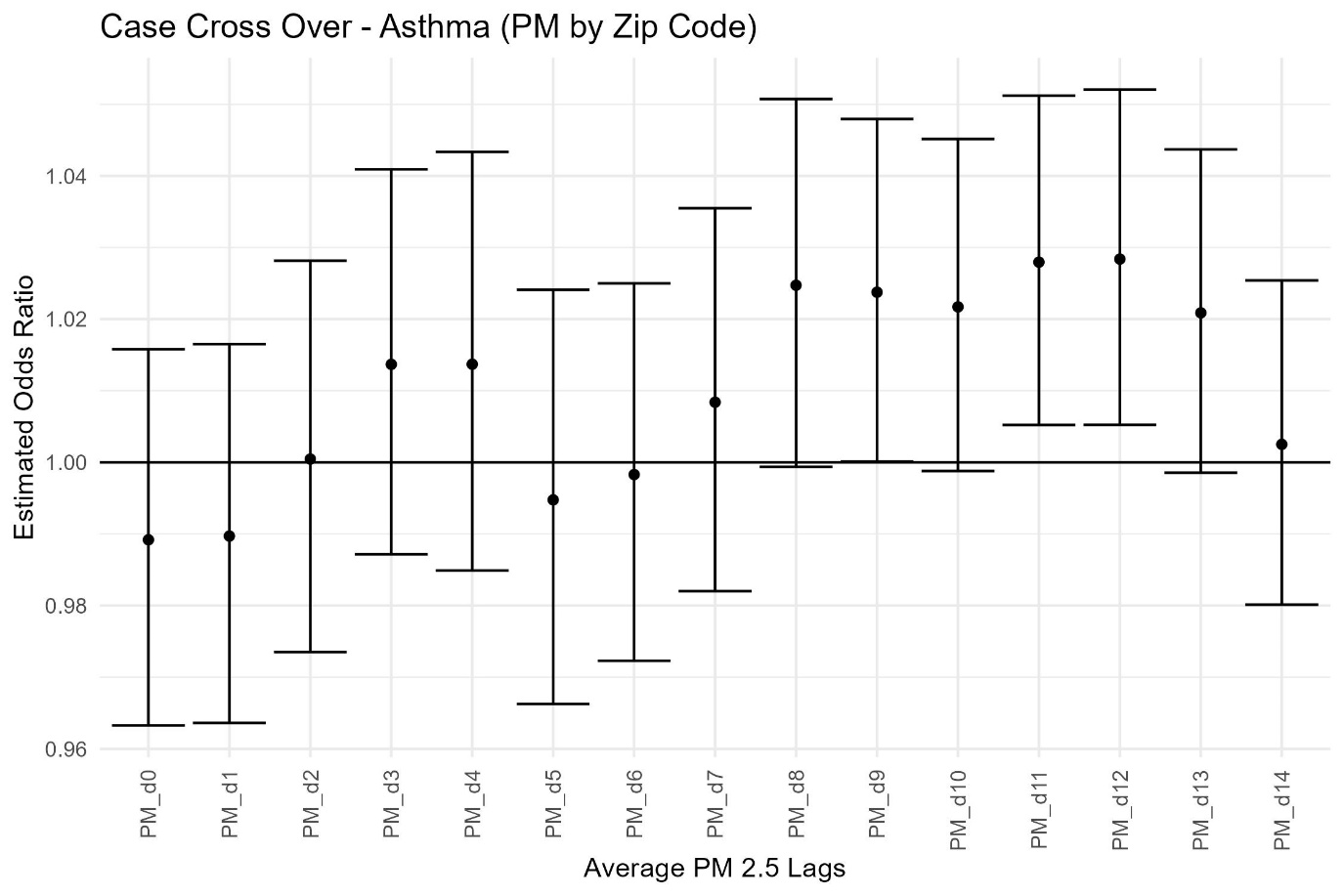
**

**(B)
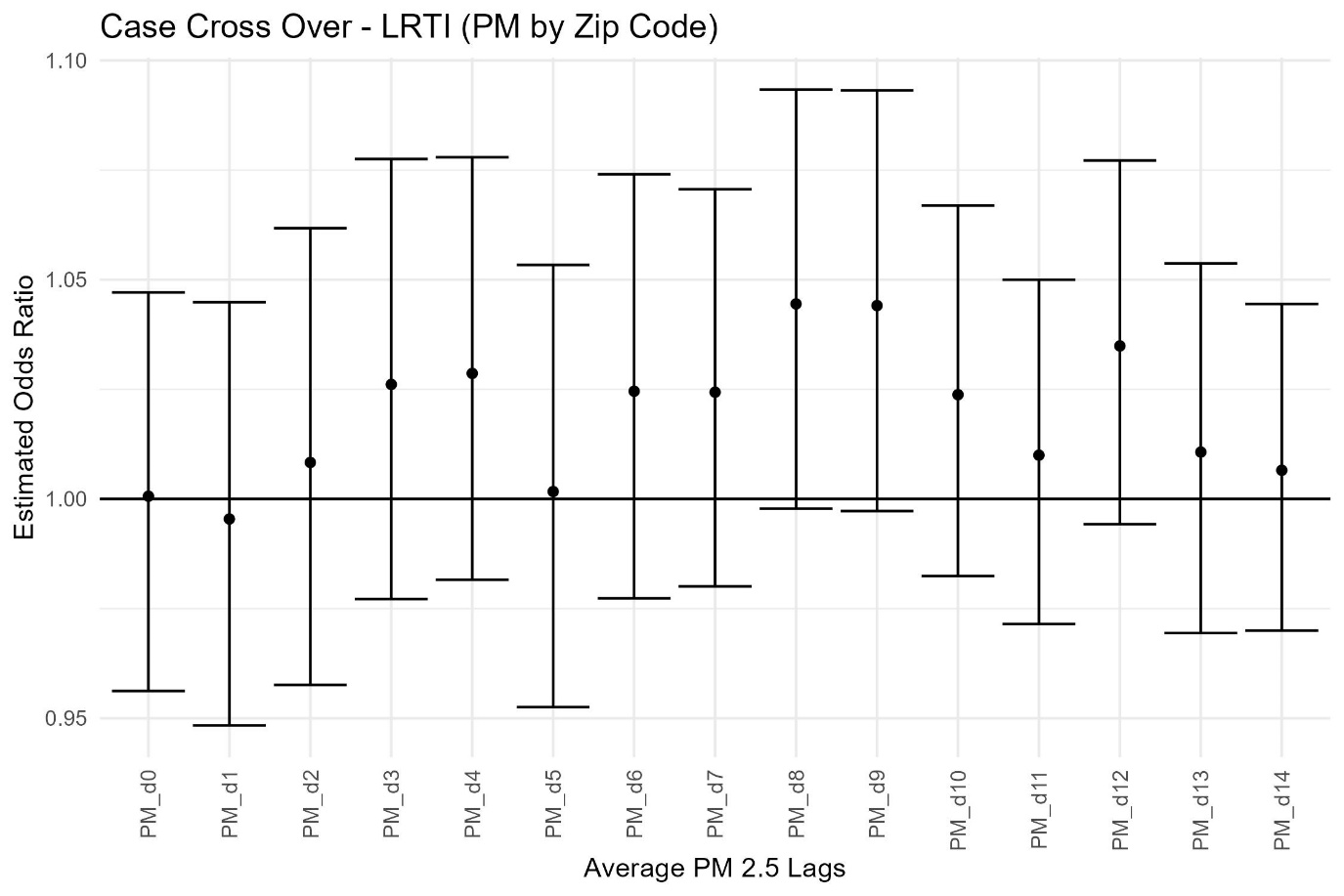
**

**(C)
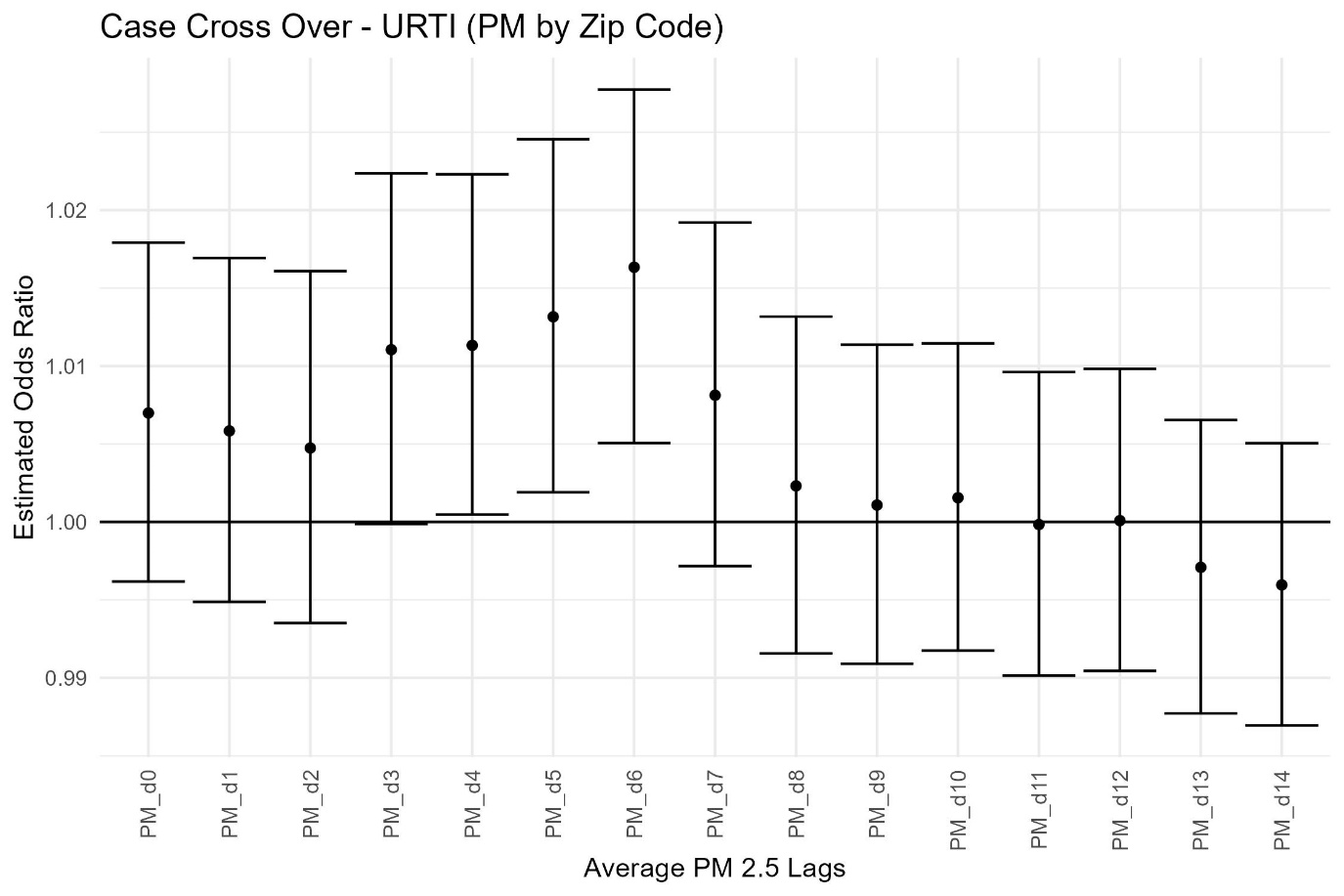
**

**Supplemental Figure 2 - Temperature lagged model comparisons.** Case-crossover datasets with distributed lag models using average weekly PM_2.5_ cumulative effect on asthma for temperature lagged single days 0 to 6 in order (A) to (G).

**(A)** Temperature single day lag 0:**
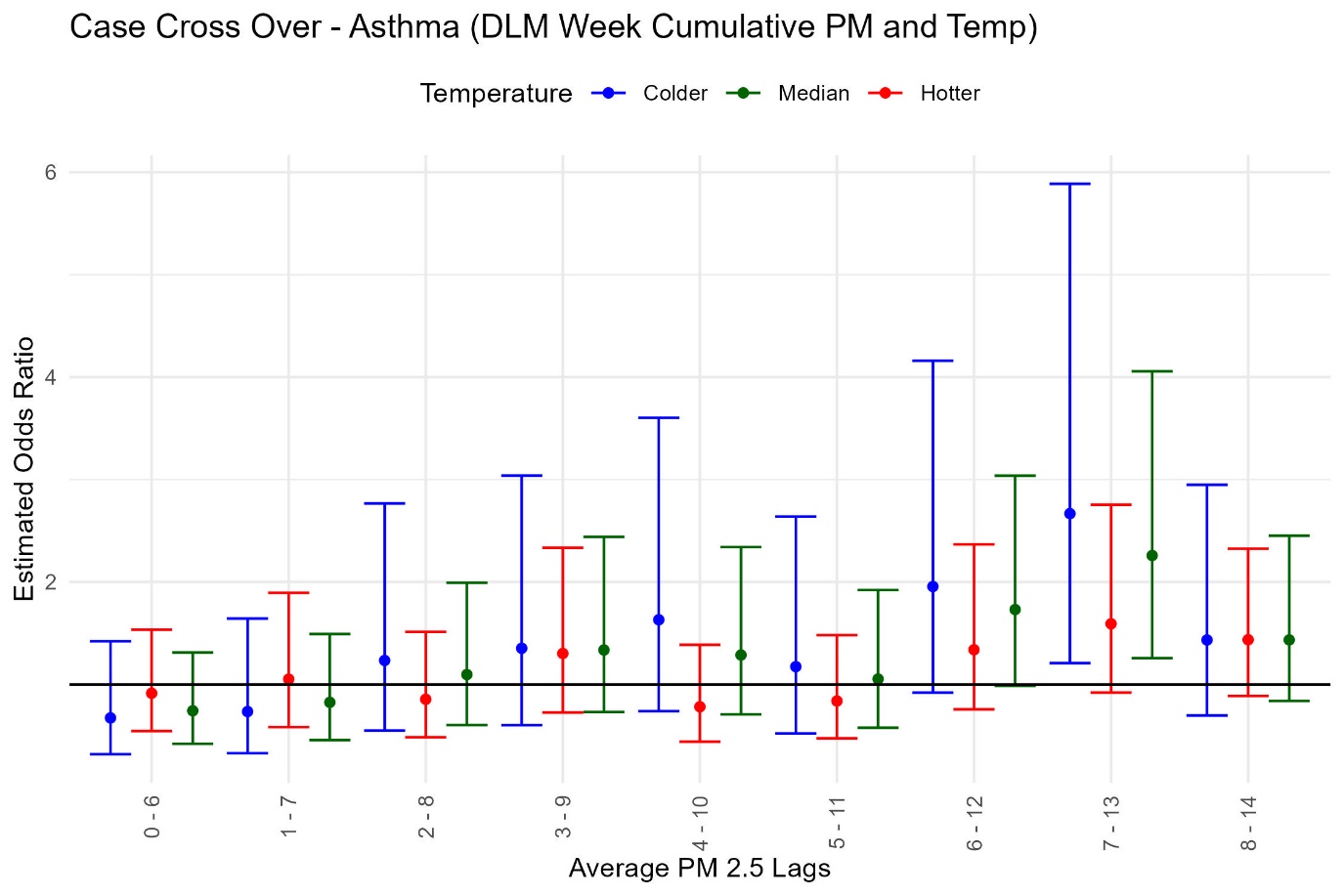
**

**(B)** Temperature single day lag 1:**
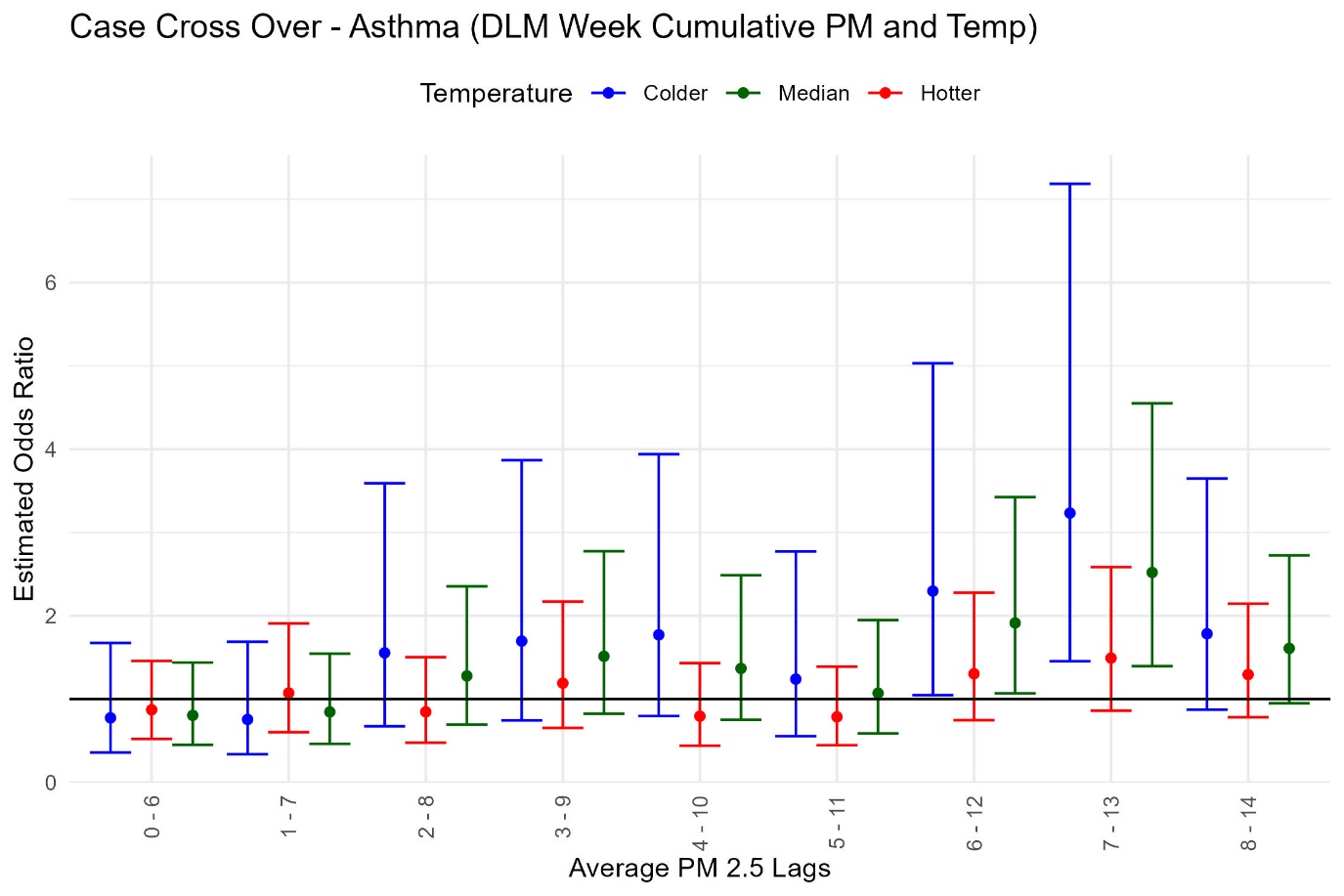
**

**(C)** Temperature single day lag 2:**
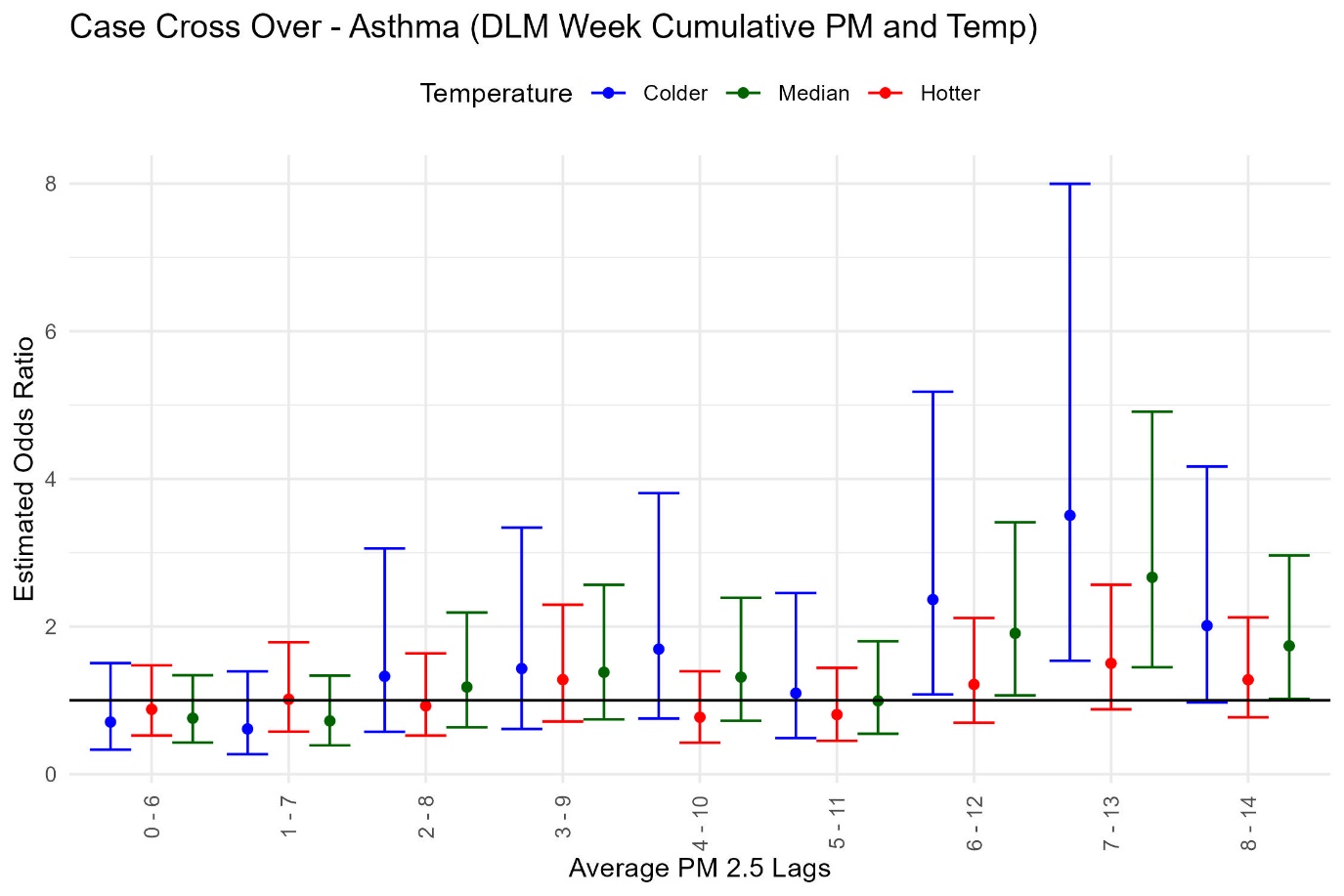
**

**(D)** Temperature single day lag 3:**
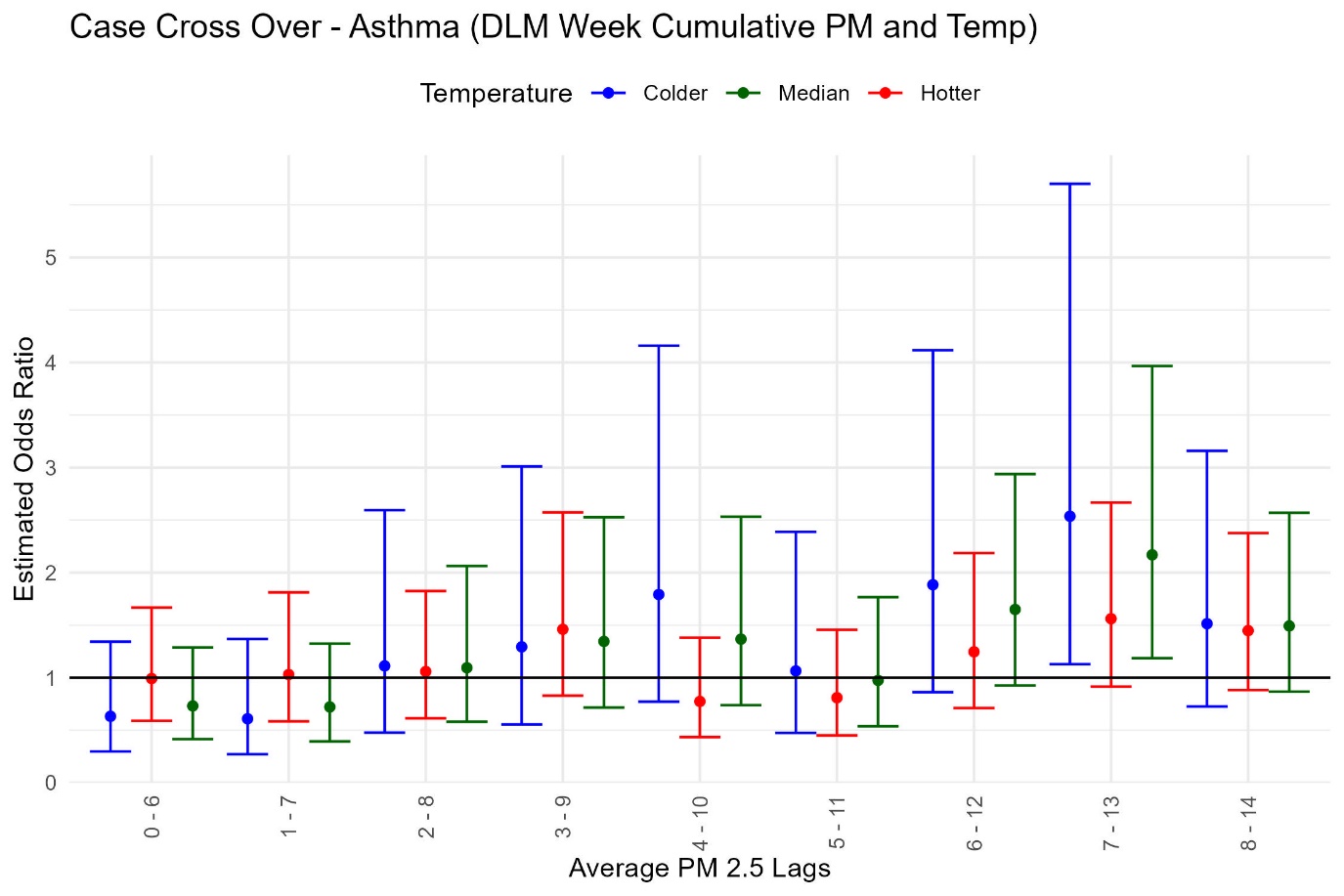
**

**(E)** Temperature single day lag 4:**
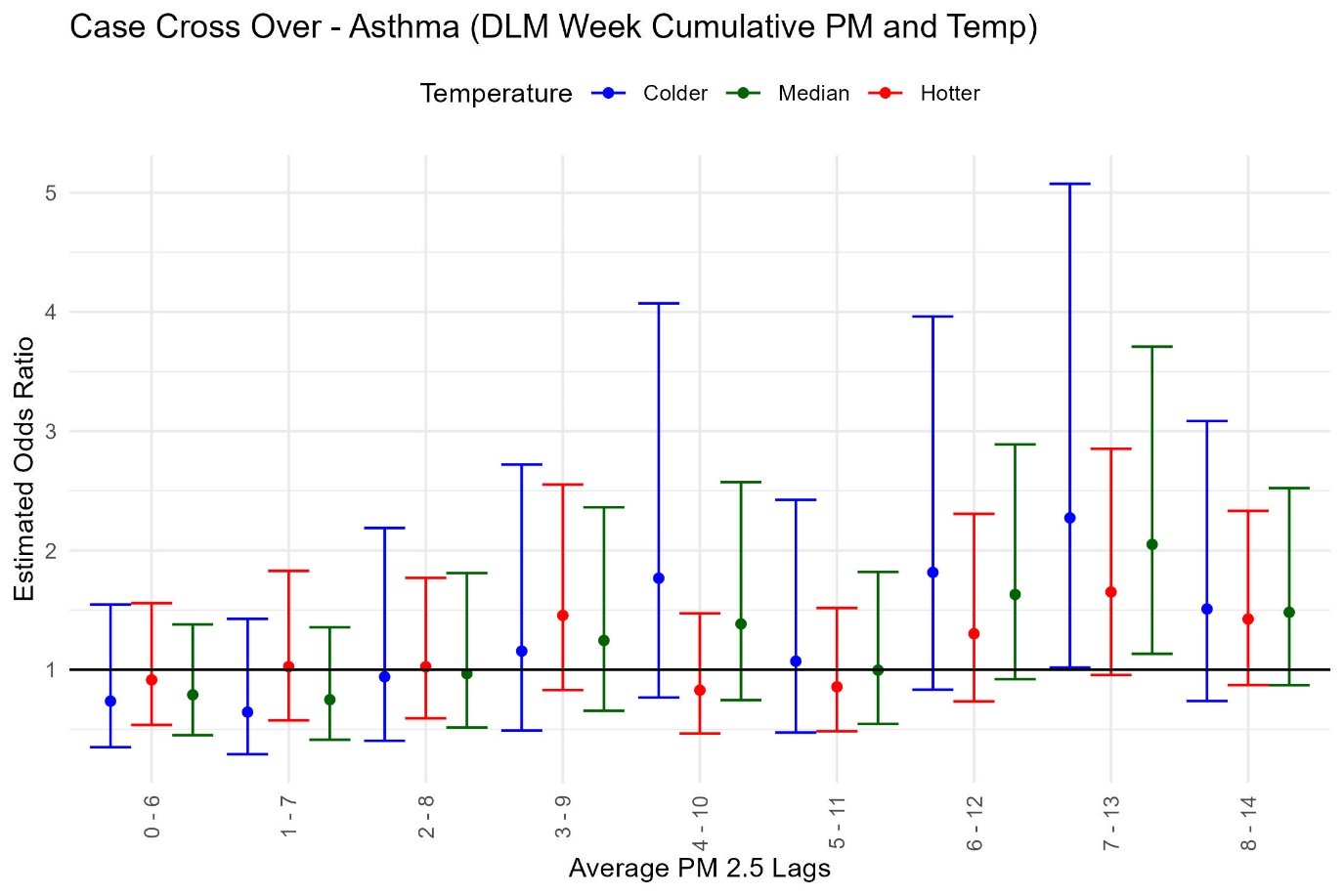
**

**(F)** Temperature single day lag 5: **
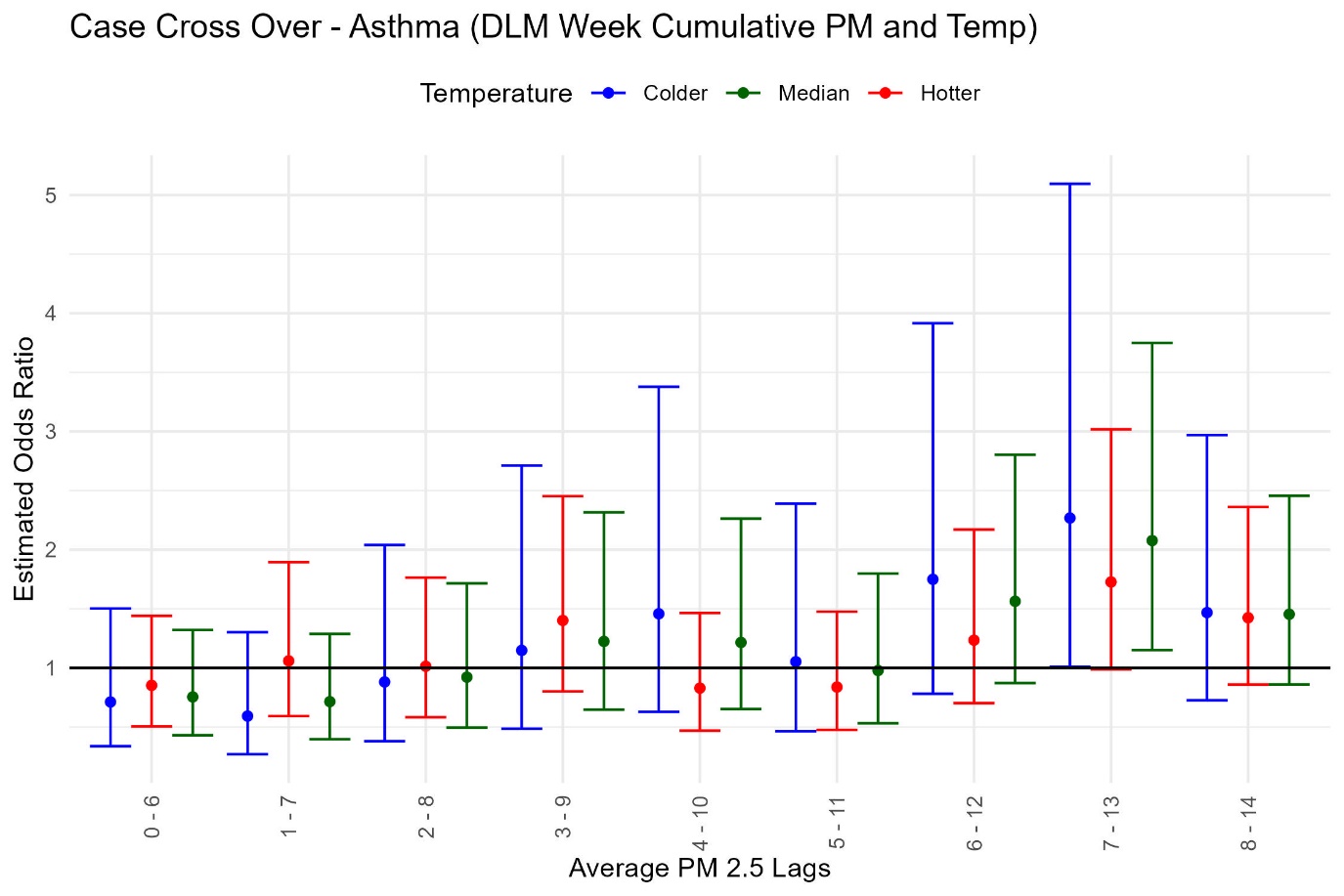
**

**(G)** Temperature single day lag 6:**
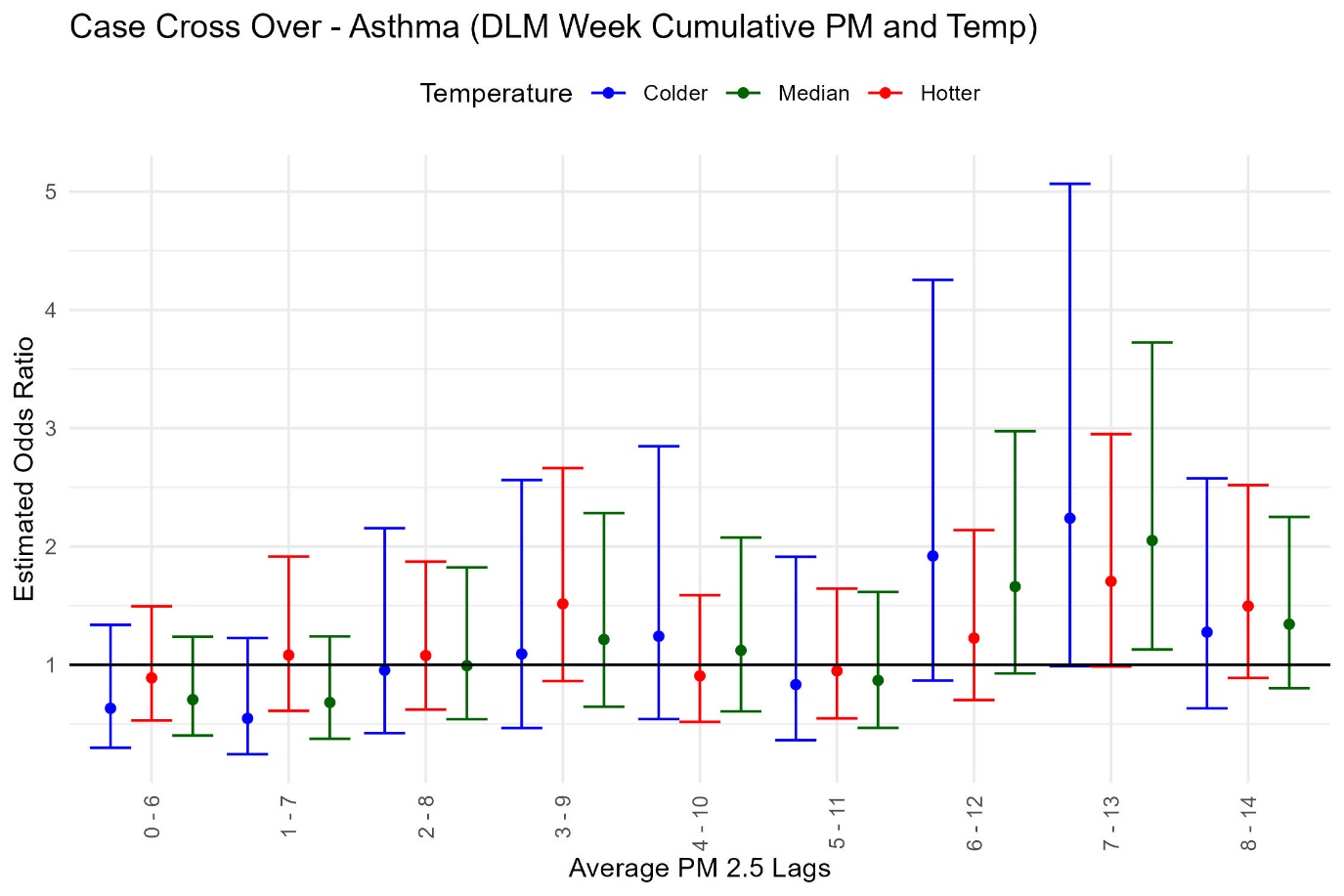
**

**Supplementary Figure 3 - Asthma and PM_2.5_**. Case cross-over results for distributed lag models of PM_2.5_ for delays of (A) single days and (B) weekly average.

**(A)**
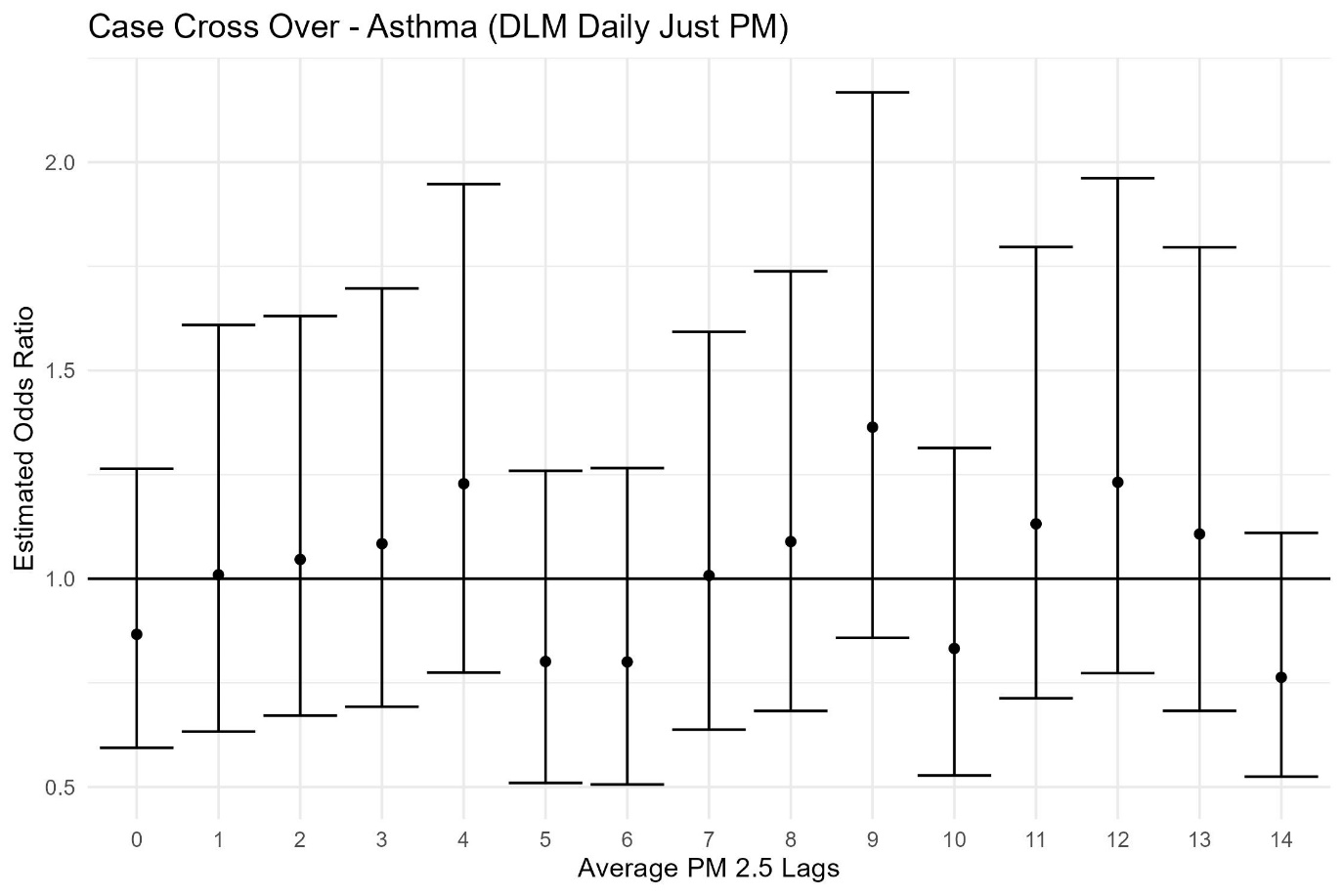


**(B)
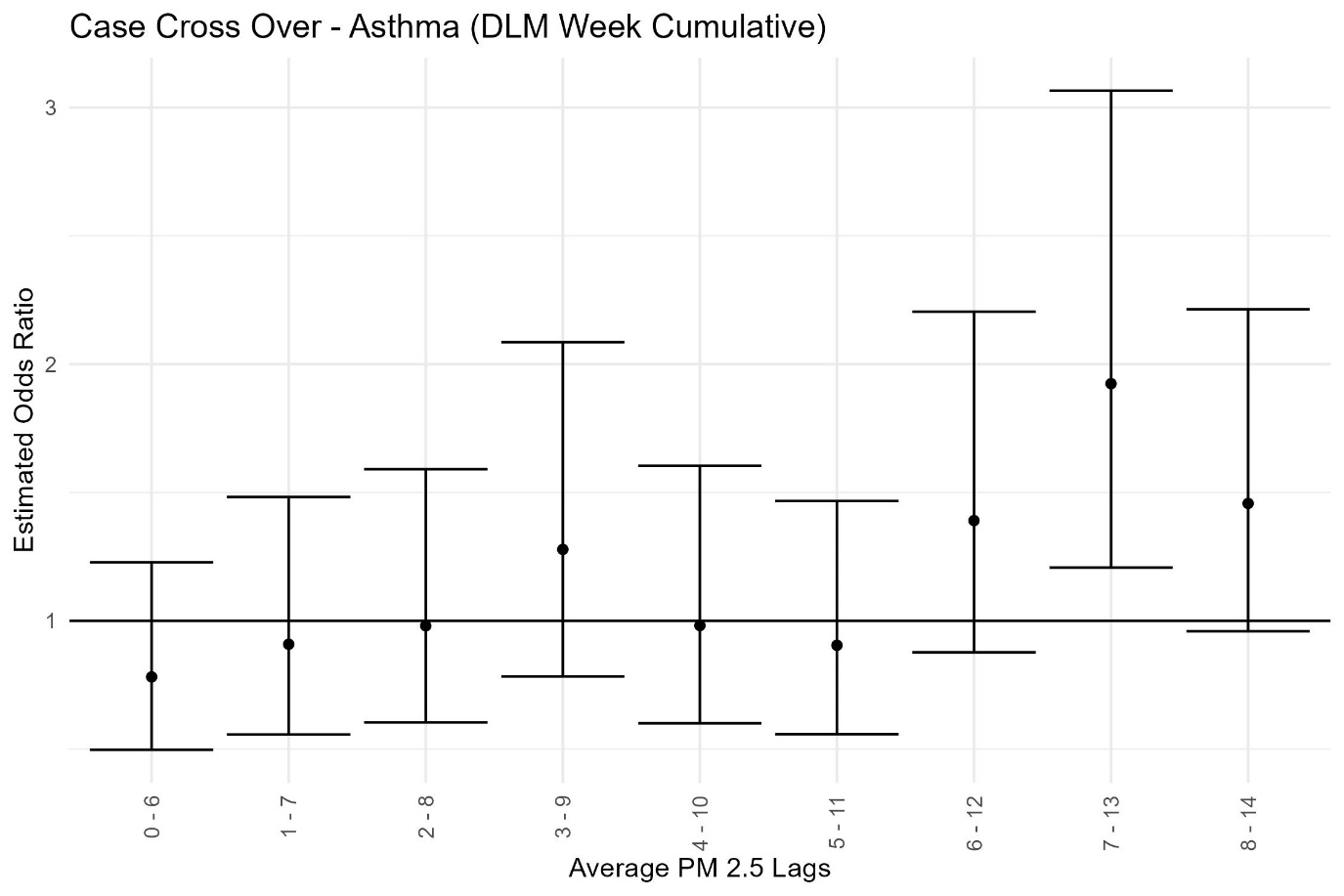
**

**Supplementary Figure 4 - Asthma and PM_2.5_ modified by temperature**. Case cross-over results for distributed lag models of PM_2.5_ for delays of (A) single days, (B) weekly average.

**(A)**
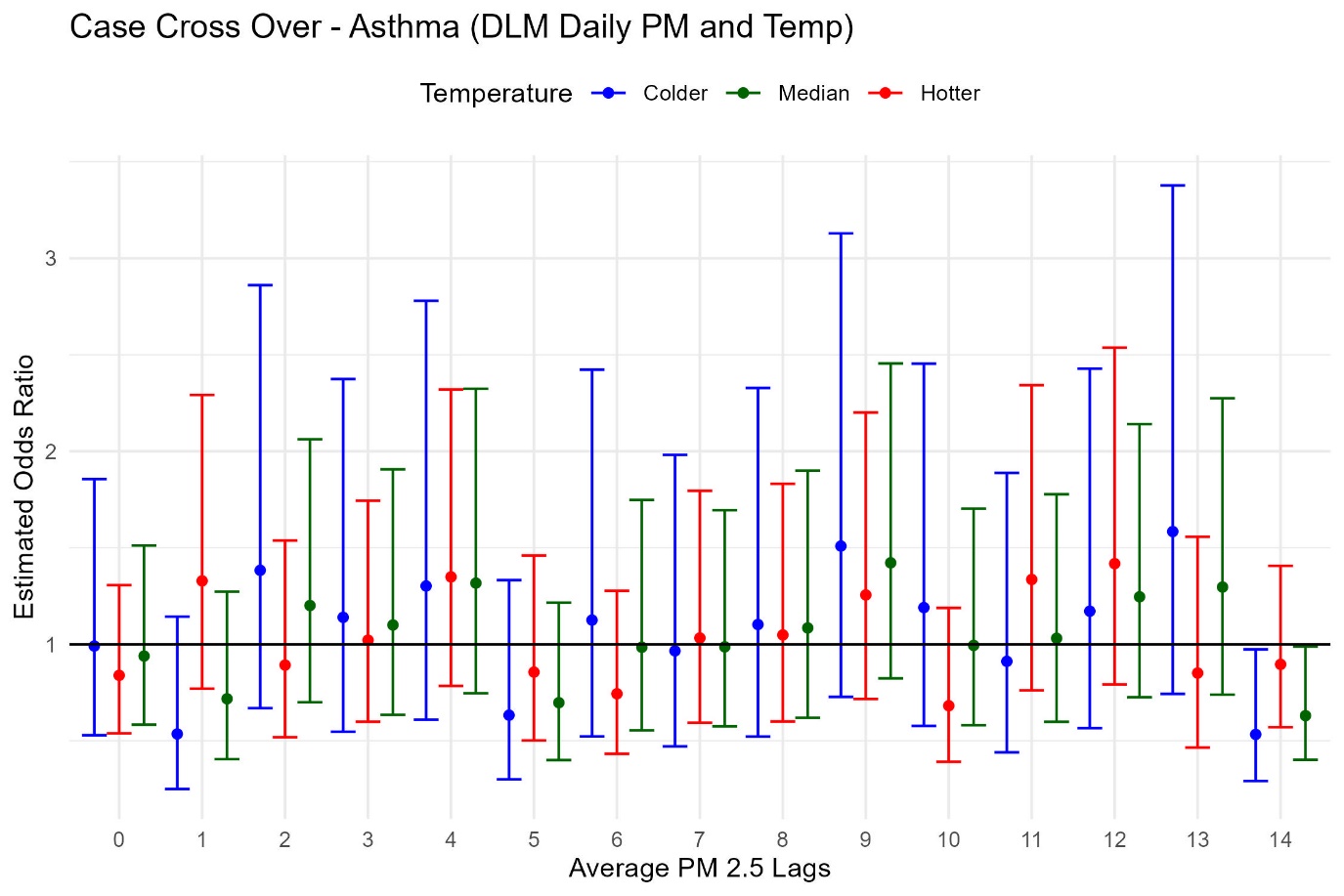


**(B)**
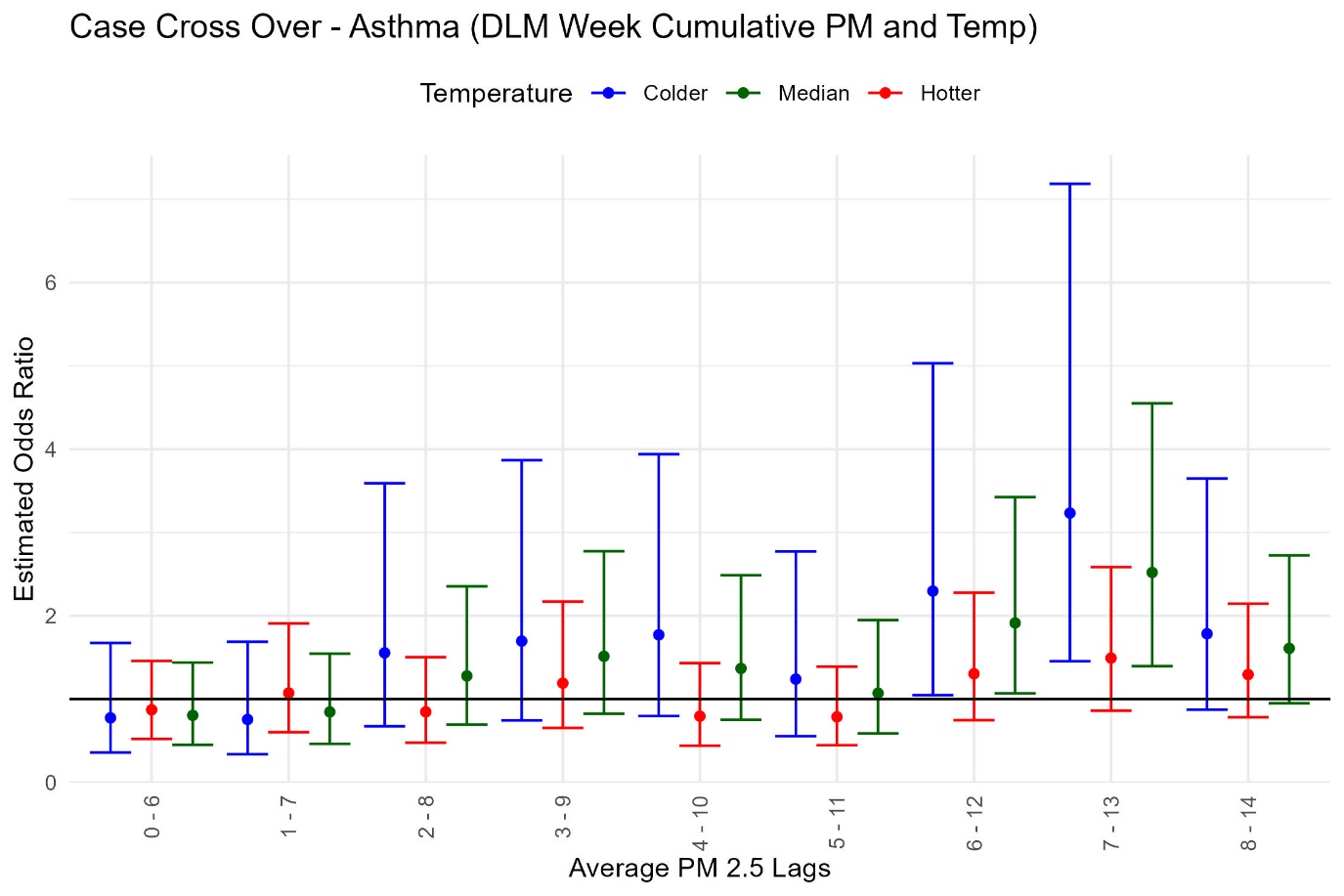


**Supplementary Figure 5 - Asthma and PM_2.5_ modified by season**. Case cross-over results for distributed lag models of PM_2.5_ for delays of (A) single days and (B) weekly average.

**(A)**
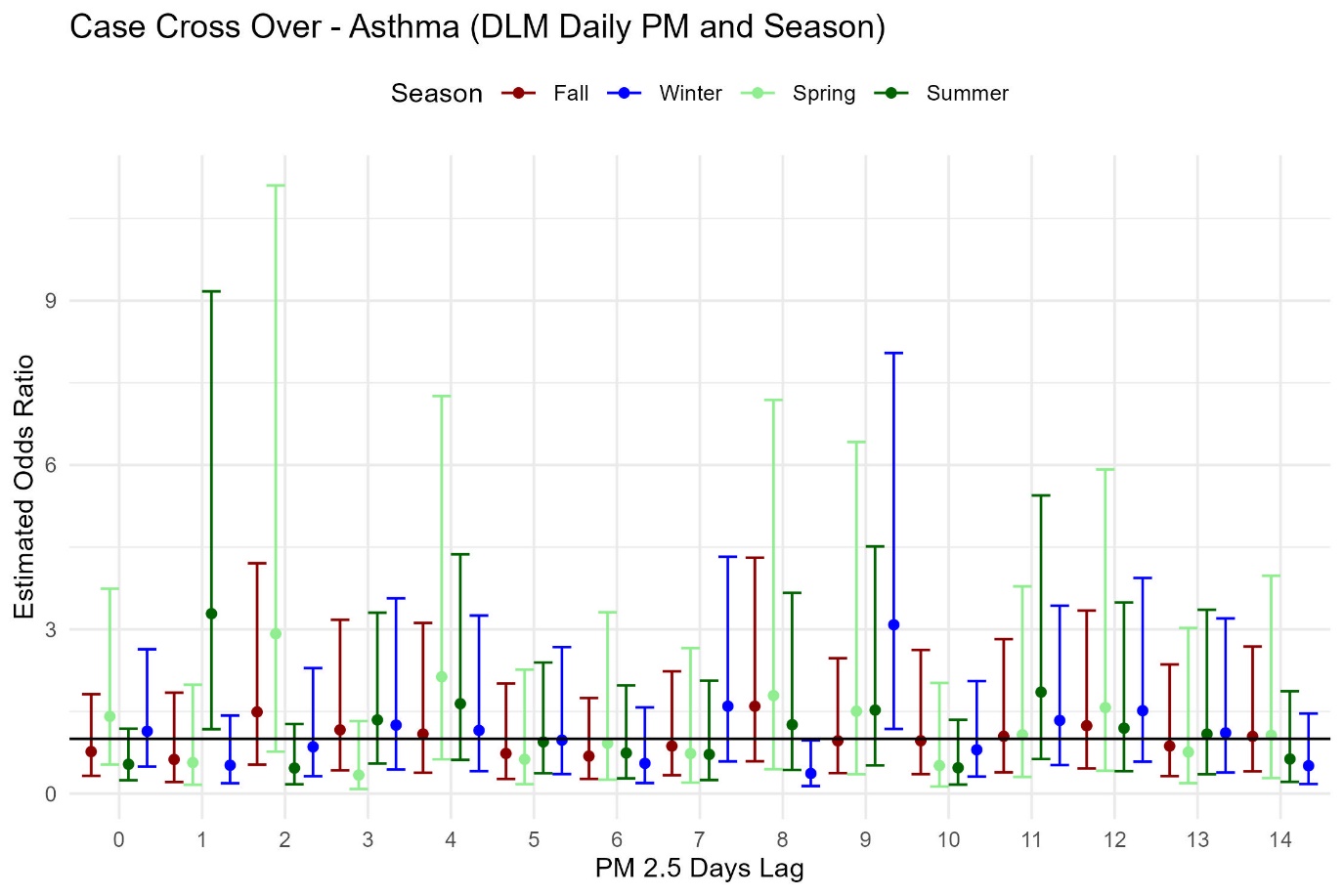


**(B)
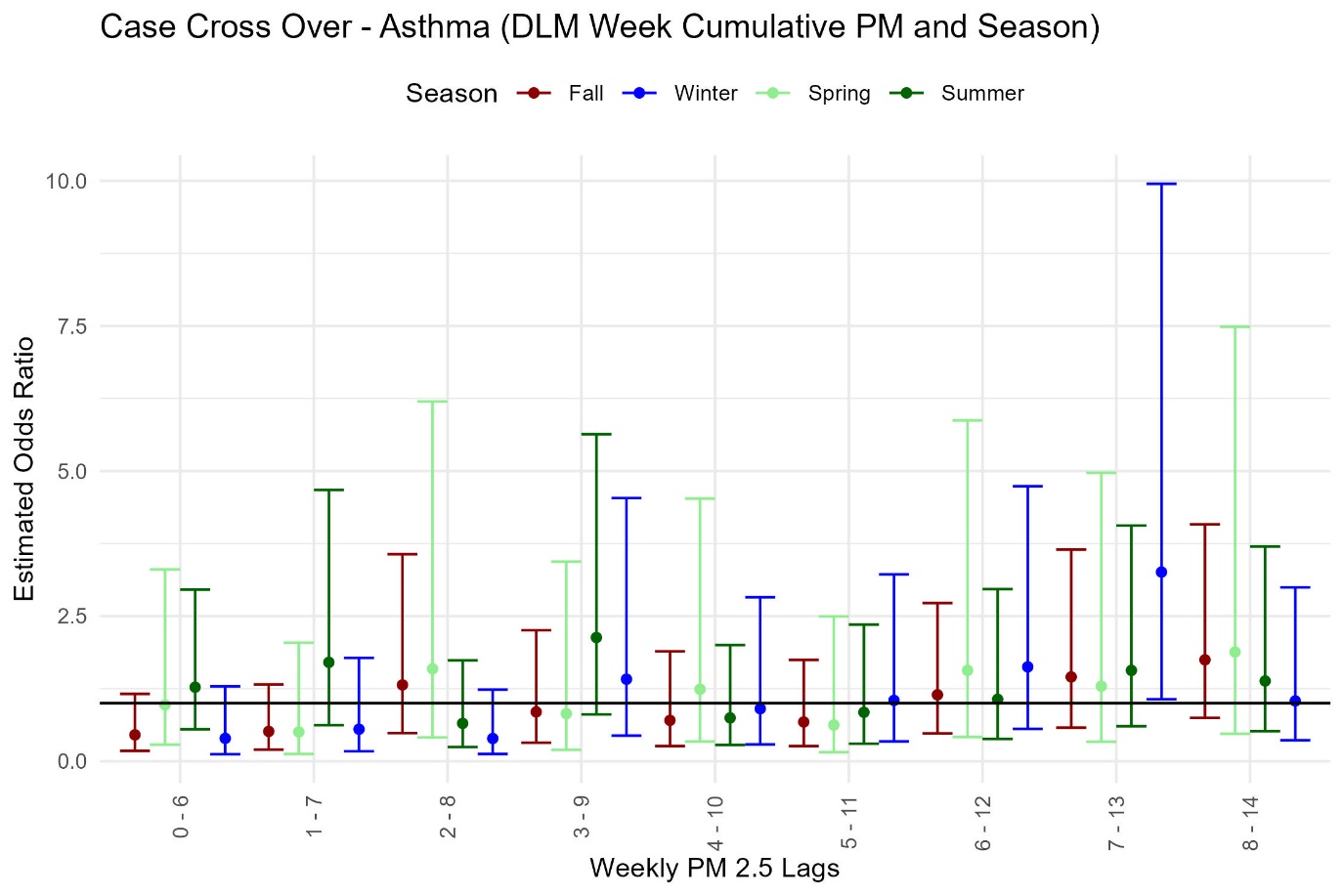
**

**Supplementary Figure 6 - LRTI and PM_2.5_**. Case cross-over results for distributed lag models of PM_2.5_ for delays of (A) single days and (B) weekly average.

**(A)**
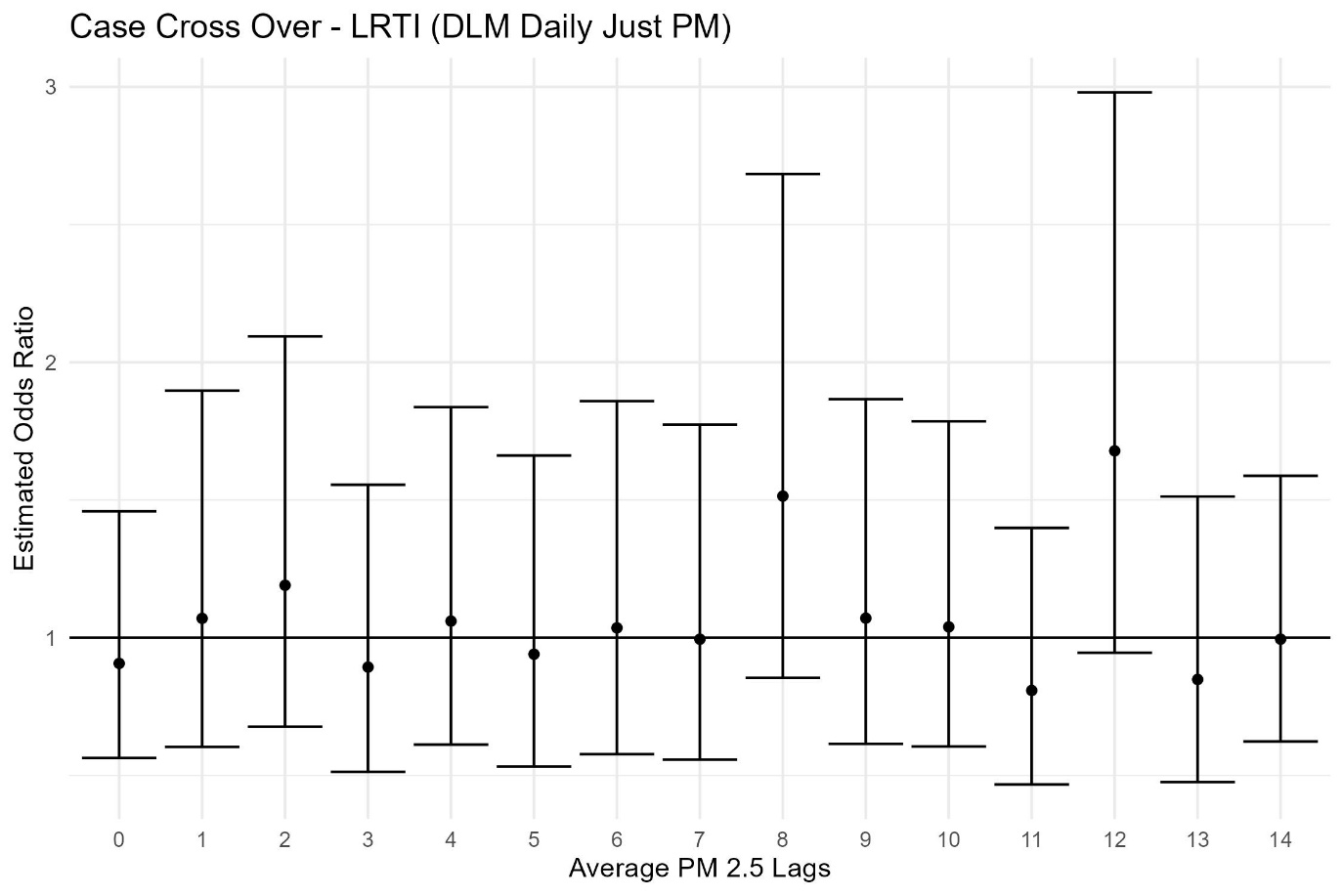


**(B)**
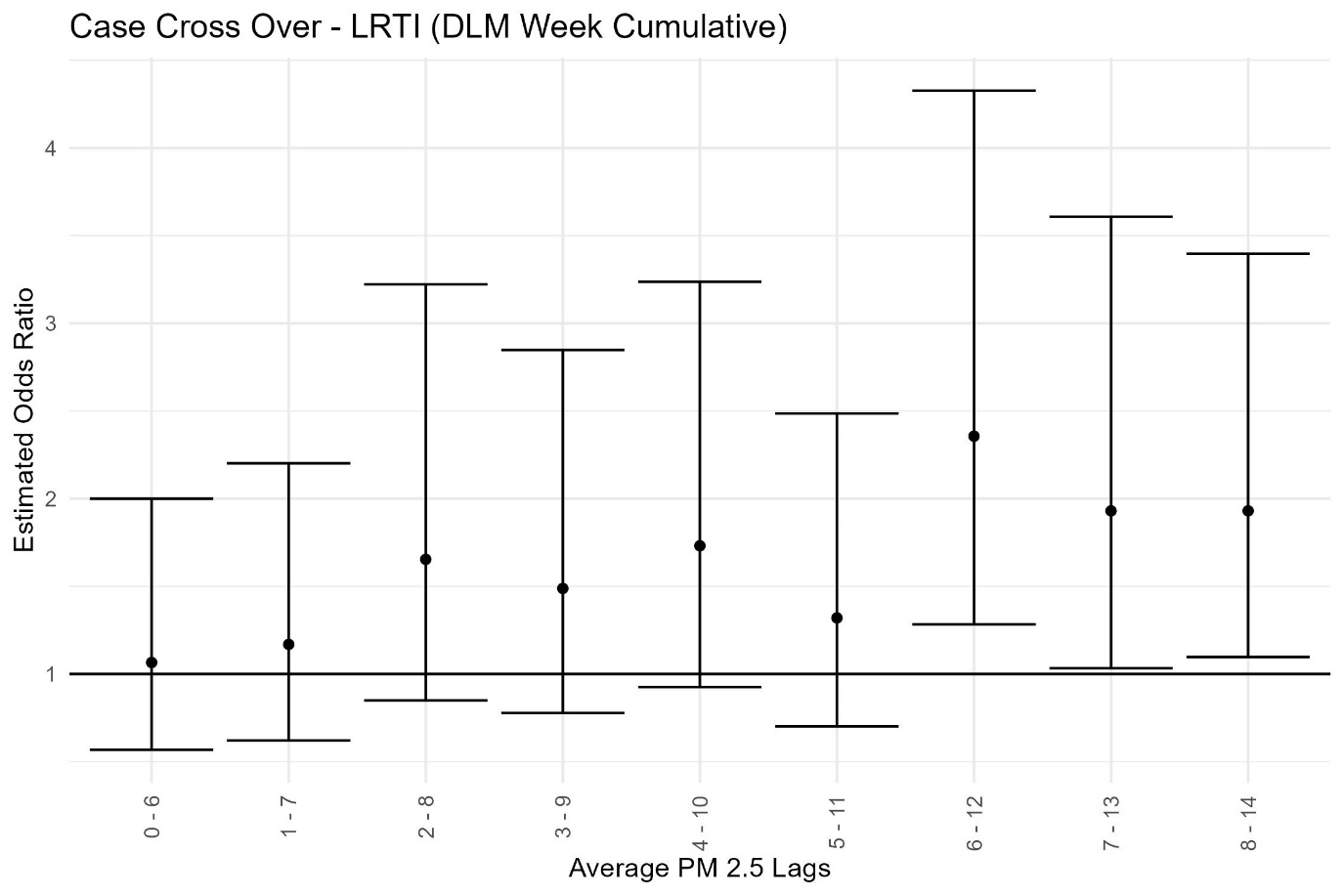


**Supplementary Figure 7 - LRTI and PM_2.5_ modified by temperature**. Case cross-over results for distributed lag models of PM_2.5_ for delays of (A) single days and (B) weekly average.

**(A)**
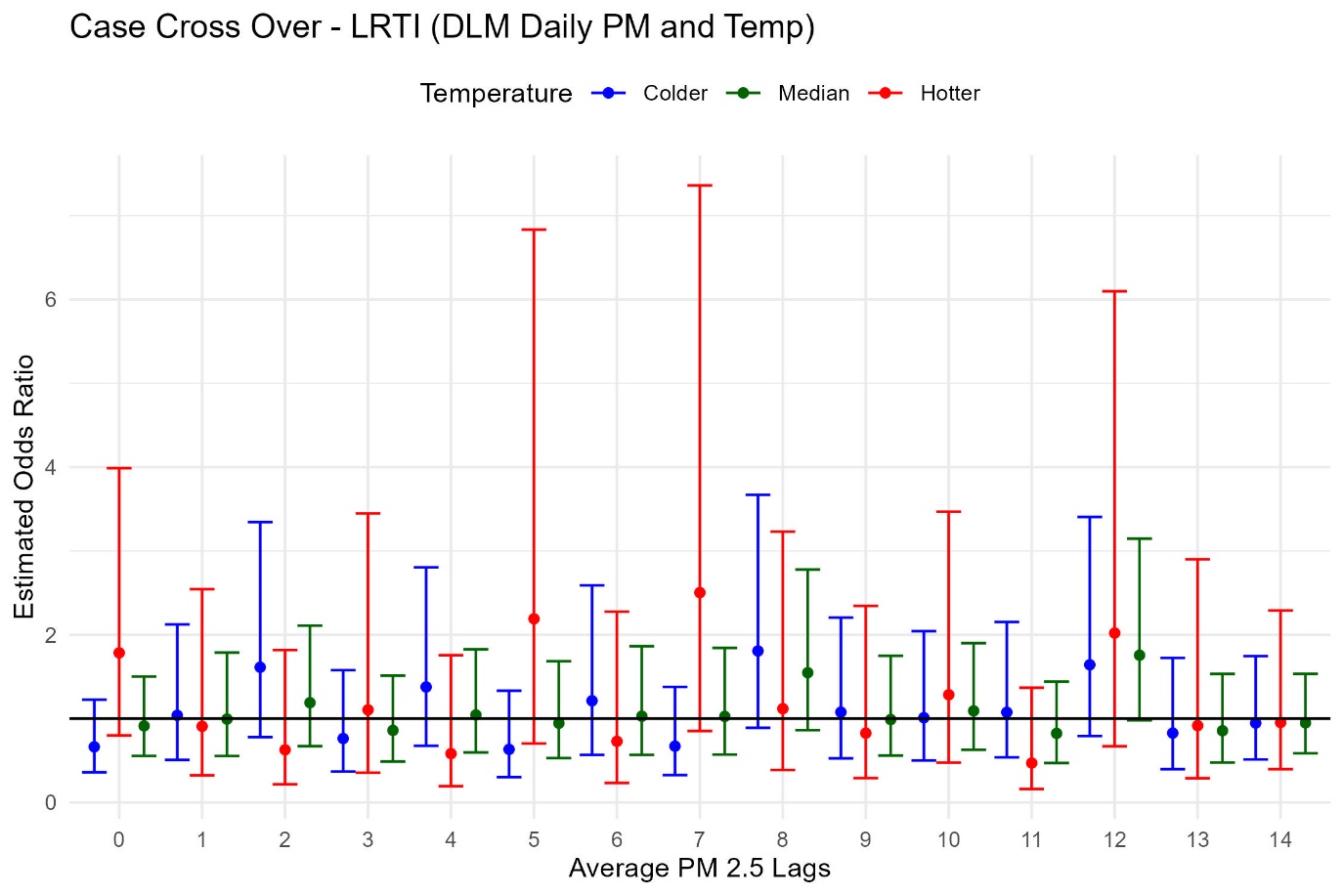


**(B)
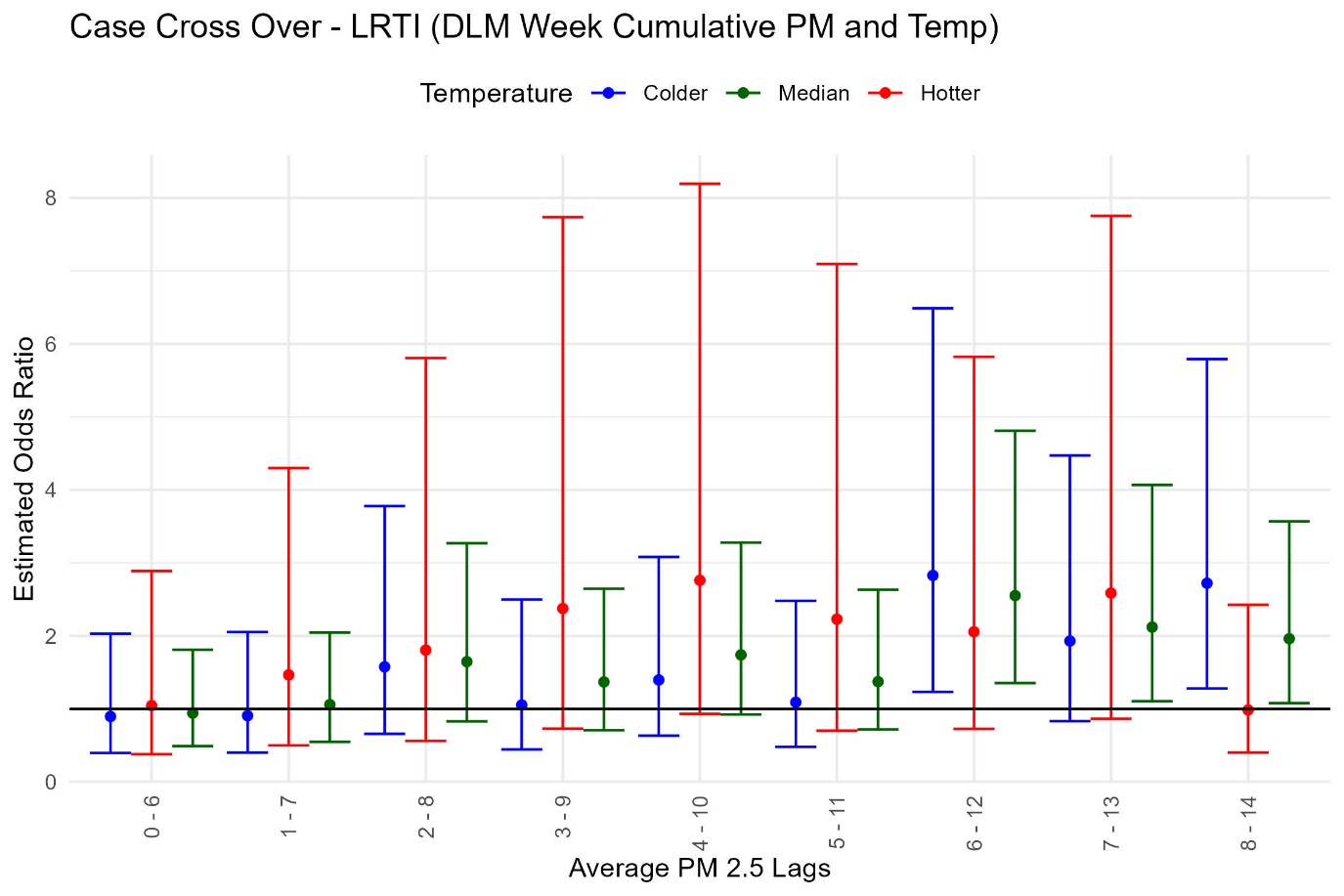
**

**Supplementary Figure 8 - LRTI and PM_2.5_ modified by season**. Case cross-over results for distributed lag models of PM_2.5_ for delays of (A) single days and (B) weekly average. Small sample sizes in these models produced unstable estimates – Y-axis cropped at 10.

**(A)**
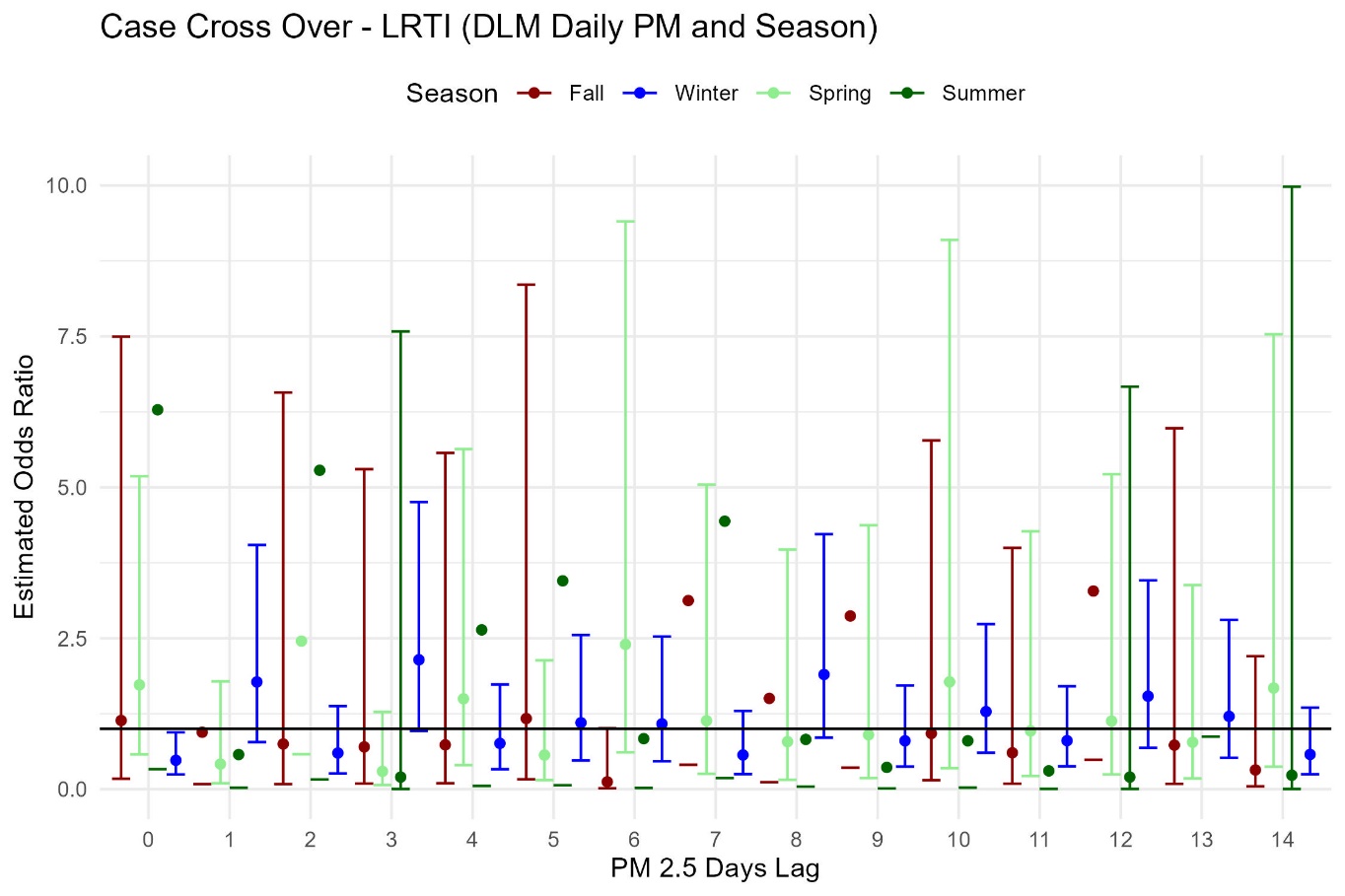


**(B)**
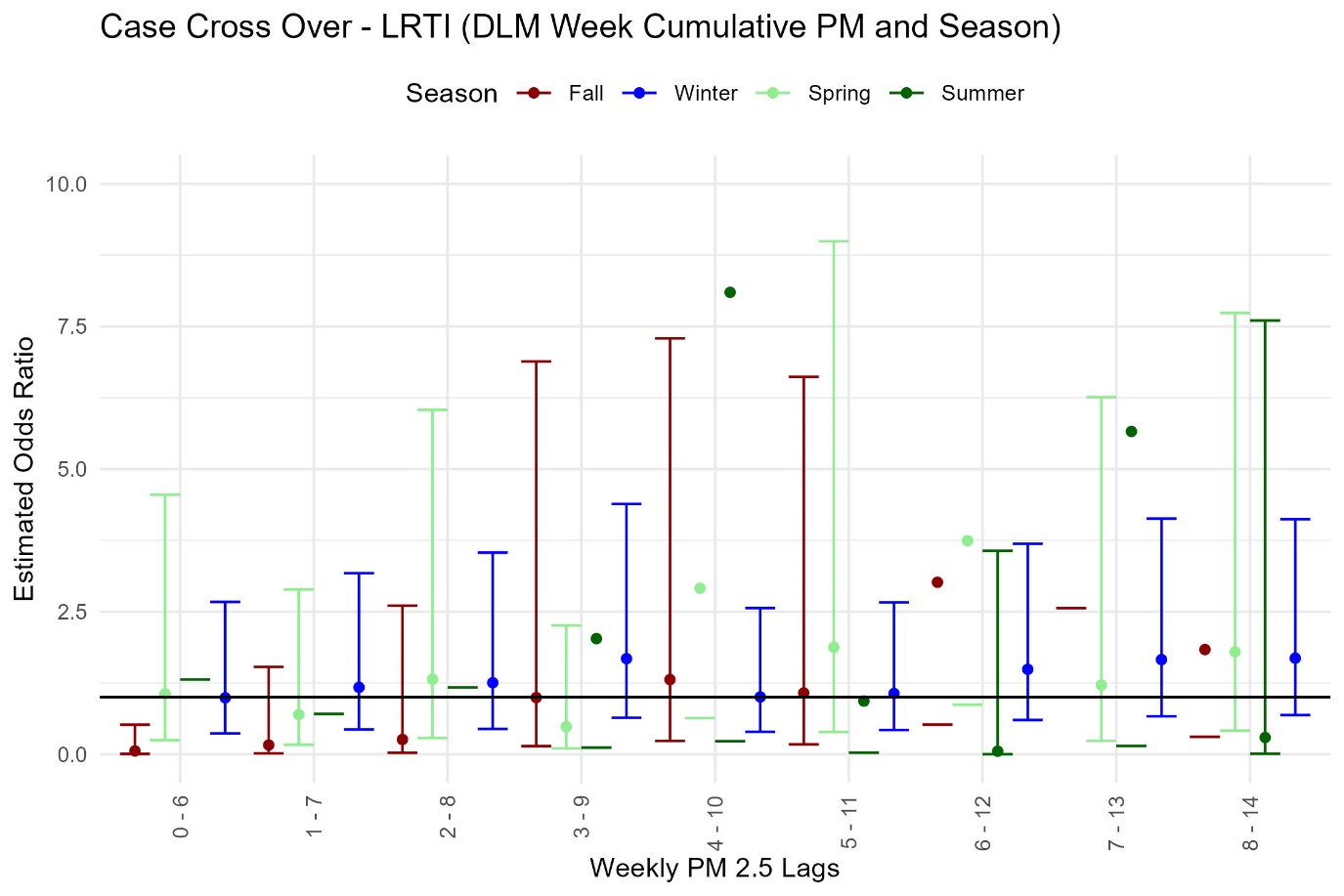


**Supplementary Figure 9 - URTI and PM_2.5_**. Case cross-over results for distributed lag models of PM_2.5_ for delays of (A) single days and (B) weekly average.

**(A)**
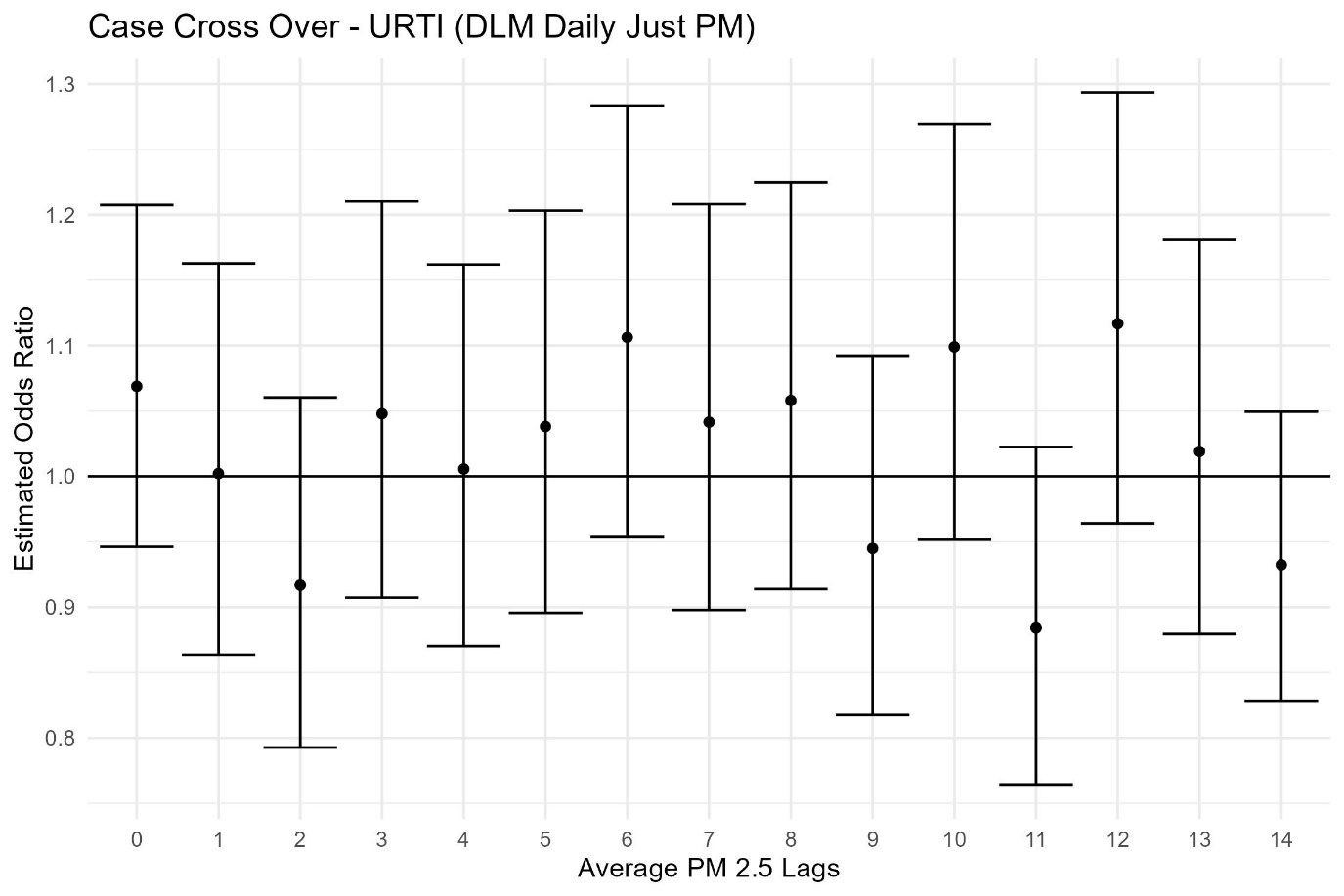


**(B)**
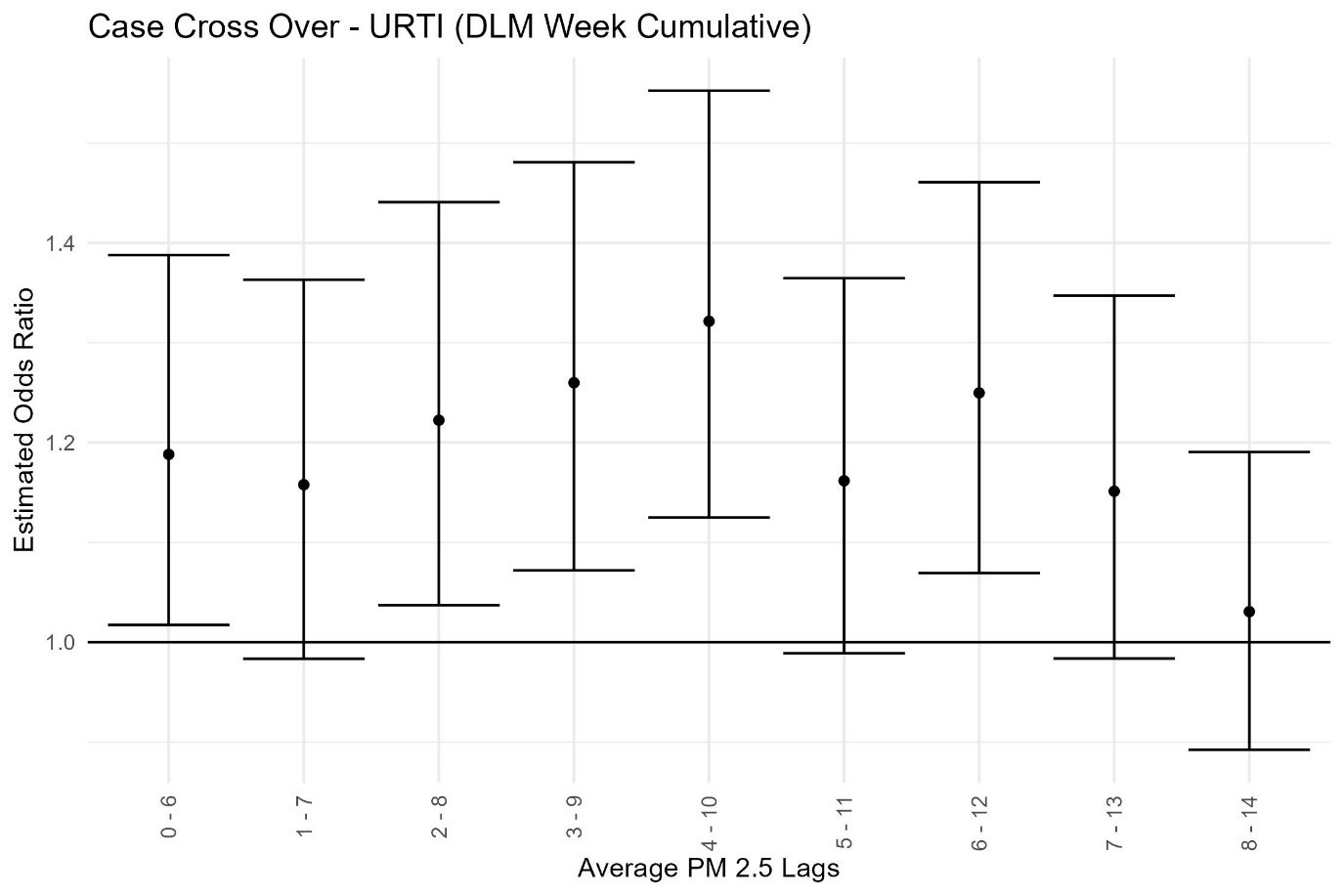


**Supplementary Figure 10 - URTI and PM_2.5_ modified by temperature**. Case cross-over results for distributed lag models of PM_2.5_ for delays of (A) single days and (B) weekly average.

**(A)**
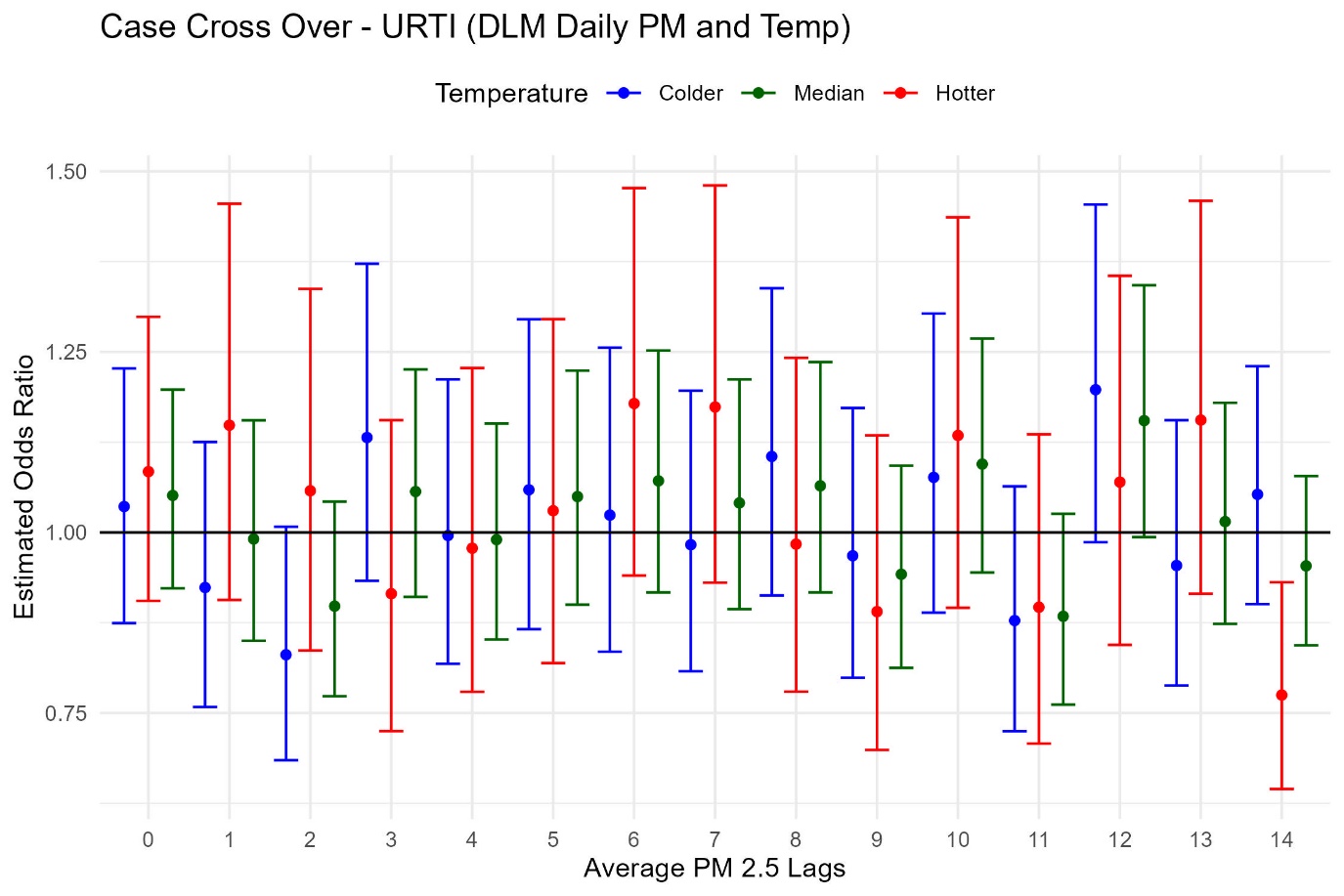


**(B)**
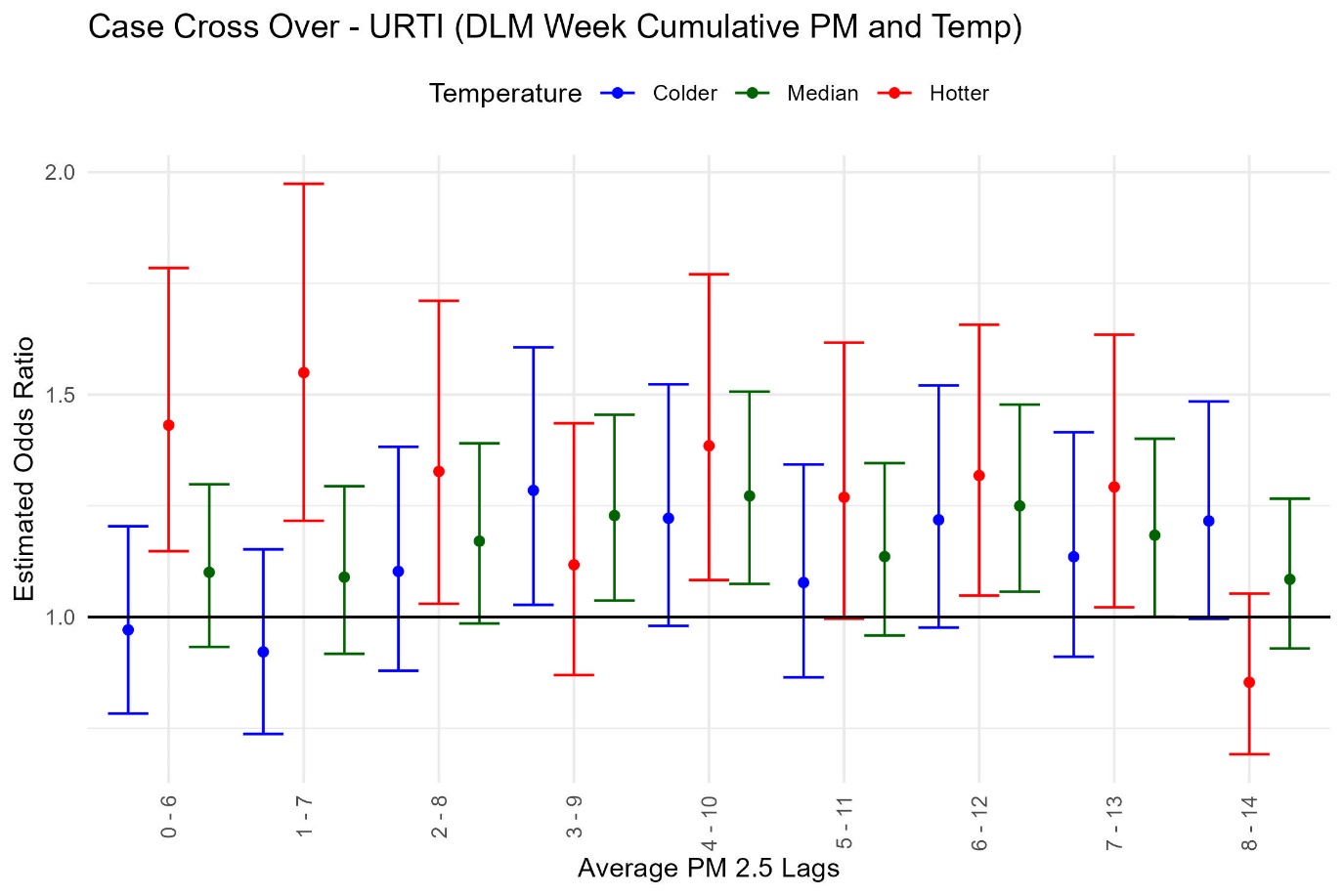


**Supplementary Figure 11 - URTI and PM_2.5_ modified by season**. Case cross-over results for distributed lag models of PM_2.5_ for delays of (A) single days and (B) weekly average.

**(A)**
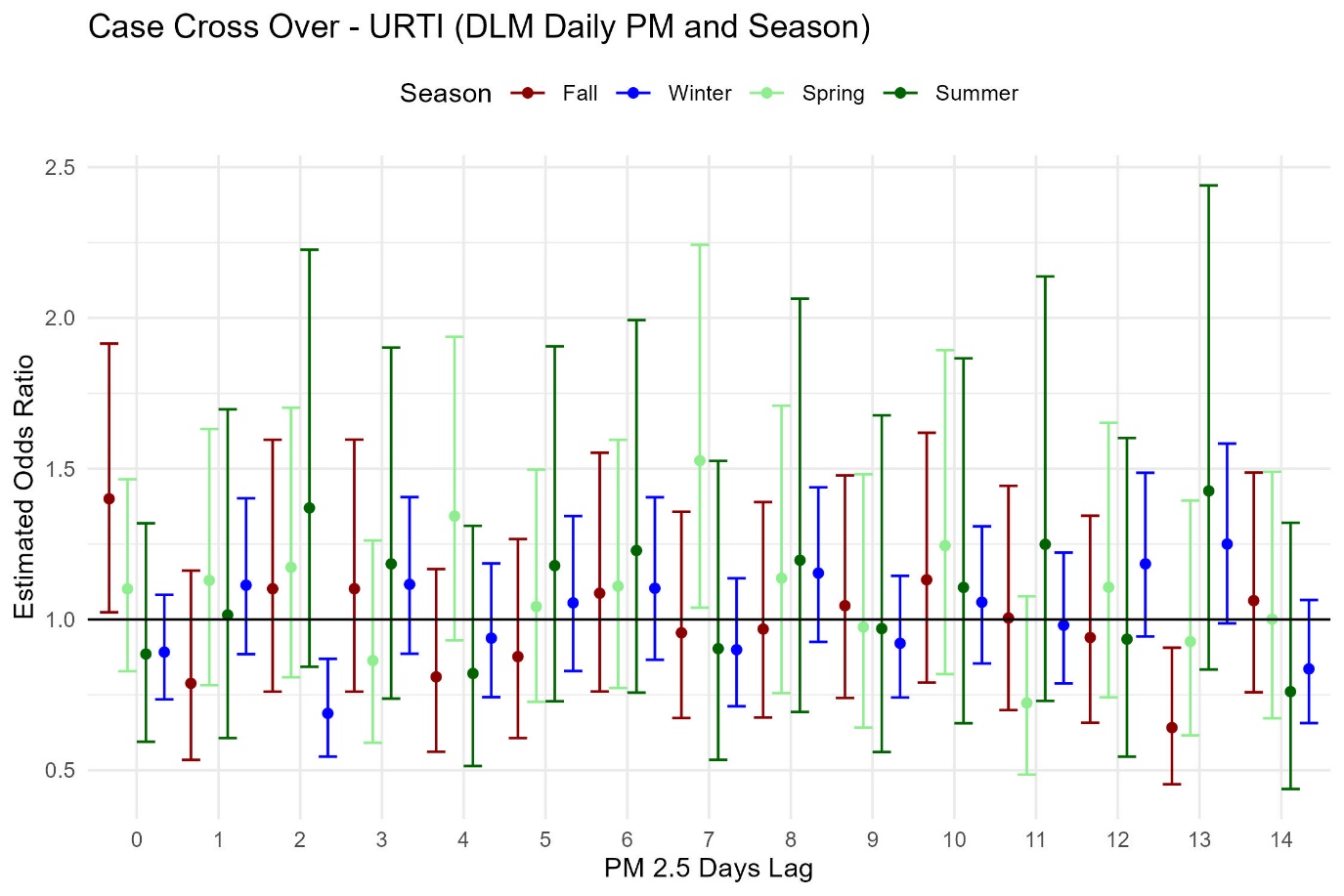


**(B)**
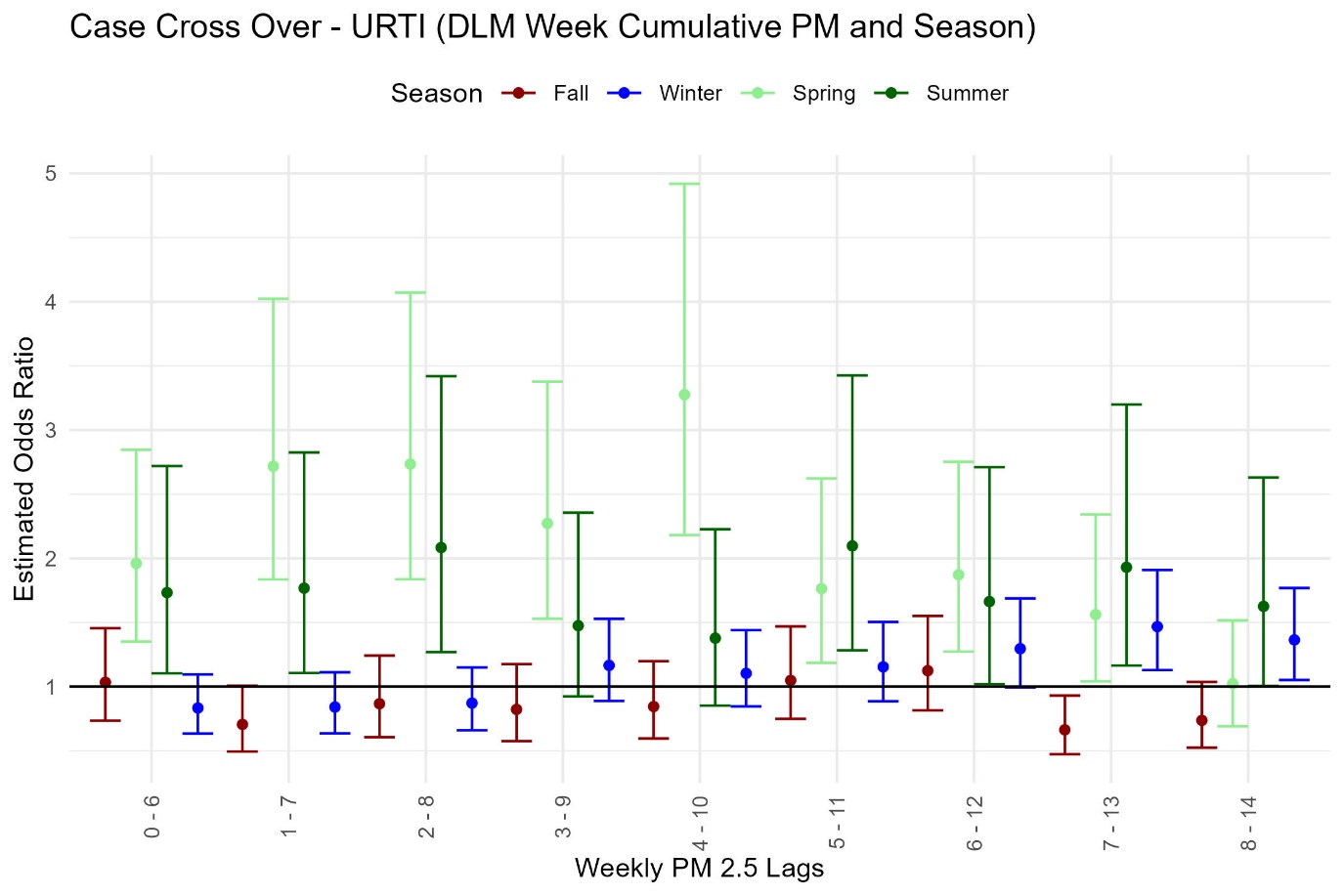

Supplement: Supplementary file 1 — Supplementary Material 1. [file 12940_2024_1082_MOESM1_ESM.docx]
